# Characterization and Tissue Tropism of Newly Identified Iflavirus and Negeviruses in *Glossina morsitans morsitans* Tsetse Flies

**DOI:** 10.3390/v13122472

**Published:** 2021-12-10

**Authors:** Irene K. Meki, Hannah-Isadora Huditz, Anton Strunov, René A. A. van der Vlugt, Henry M. Kariithi, Mohammadreza Rezapanah, Wolfgang J. Miller, Just M. Vlak, Monique M. van Oers, Adly M. M. Abd-Alla

**Affiliations:** 1 Insect Pest Control Laboratory, Joint FAO/IAEA Programme of Nuclear Techniques in Food and Agriculture, International Atomic Energy Agency, Vienna International Centre, P.O. Box 100, 1400 Vienna, Austria; i.meki@iaea.org (I.K.M.); h.huditz@iaea.org (H.-I.H.); henri.muriuki@gmail.com (H.M.K.); 2 Laboratory of Virology, Wageningen University and Research, 6708 PB Wageningen, The Netherlands; rene.vandervlugt@wur.nl (R.A.A.v.d.V.); justvlak1@gmail.com (J.M.V.); monique.vanoers@wur.nl (M.M.v.O.); 3 Lab Genome Dynamics, Department Cell & Developmental Biology, Center for Anatomy and Cell Biology, Medical University of Vienna, Schwarzspanierstraße 17, 1090 Vienna, Austria; anton.strunov@meduniwien.ac.at (A.S.); wolfgang.miller@meduniwien.ac.at (W.J.M.); 4 Southeast Poultry Research Laboratory, U.S. National Poultry Research Center, Agricultural Research Service, USDA-ARS, Athens, GA 30605, USA; 5 Biotechnology Research Center, Kenya Agricultural and Livestock Research Organization, Nairobi P.O. Box 57811-00200, Kenya; 6 Iranian Research Institute of Plant Protection (IRIPP), Agricultural Research Education and Extension Organization (AREEO), Tehran 19395, Iran; rezapana@yahoo.com

**Keywords:** *Iflaviridae*, sterile insect technique, mass rearing, RNA viruses, FISH, stellaris probes

## Abstract

Tsetse flies cause major health and economic problems as they transmit trypanosomes causing sleeping sickness in humans (Human African Trypanosomosis, HAT) and nagana in animals (African Animal Trypanosomosis, AAT). A solution to control the spread of these flies and their associated diseases is the implementation of the Sterile Insect Technique (SIT). For successful application of SIT, it is important to establish and maintain healthy insect colonies and produce flies with competitive fitness. However, mass production of tsetse is threatened by covert virus infections, such as the Glossina pallidipes salivary gland hypertrophy virus (GpSGHV). This virus infection can switch from a covert asymptomatic to an overt symptomatic state and cause the collapse of an entire fly colony. Although the effects of GpSGHV infections can be mitigated, the presence of other covert viruses threaten tsetse mass production. Here we demonstrated the presence of two single-stranded RNA viruses isolated from *Glossina morsitans morsitans* originating from a colony at the Seibersdorf rearing facility. The genome organization and the phylogenetic analysis based on the RNA-dependent RNA polymerase (RdRp) revealed that the two viruses belong to the genera *Iflavirus* and *Negevirus*, respectively. The names proposed for the two viruses are Glossina morsitans morsitans iflavirus (GmmIV) and Glossina morsitans morsitans negevirus (GmmNegeV). The GmmIV genome is 9685 nucleotides long with a poly(A) tail and encodes a single polyprotein processed into structural and non-structural viral proteins. The GmmNegeV genome consists of 8140 nucleotides and contains two major overlapping open reading frames (ORF1 and ORF2). ORF1 encodes the largest protein which includes a methyltransferase domain, a ribosomal RNA methyltransferase domain, a helicase domain and a RdRp domain. In this study, a selective RT-qPCR assay to detect the presence of the negative RNA strand for both GmmIV and GmmNegeV viruses proved that both viruses replicate in *G. m. morsitans*. We analyzed the tissue tropism of these viruses in *G. m. morsitans* by RNA-FISH to decipher their mode of transmission. Our results demonstrate that both viruses can be found not only in the host’s brain and fat bodies but also in their reproductive organs, and in milk and salivary glands. These findings suggest a potential horizontal viral transmission during feeding and/or a vertically viral transmission from parent to offspring. Although the impact of GmmIV and GmmNegeV in tsetse rearing facilities is still unknown, none of the currently infected tsetse species show any signs of disease from these viruses.

## 1. Introduction

Tsetse flies are vectors of trypanosomes, causative agents of sleeping sickness in humans and nagana in livestock. Although only 8–10 tsetse species are of medical and/or economic importance, there are about 31 tsetse species and sub-species distributed in 37 sub-Saharan African countries [1]. To date, the only virus known to be associated with tsetse flies is a large, rod-shaped dsDNA virus, Glossina pallidipes salivary gland hypertrophy virus (GpSGHV; family *Hytrosaviridae*) [2]. GpSGHV symptomatic infections are known to cause reproductive dysfunctions, which can result in the collapse of the fly colonies [3,4]. This jeopardizes the control of tsetse (vector) and trypanosomosis through the application of the Sterile Insect Technique (SIT), a birth control technique that requires mass release of sterile males with competitive fitness into the target insect population to mate with wild virgin females resulting in the lack of offspring [5,6].

In addition to GpSGHV, tsetse flies also harbor symbiotic bacteria—*Wigglesworthia glossinidia*, *Sodalis glossinidius*, *Wolbachia pipientis* and *Spiroplasma*—which may contribute to the digestion of nutrients in the flies as well as regulate fly reproduction and immunity. These findings indicate interdependence of the symbionts and GpSGHV which may influence GpSGHV transmission and pathologies [7,8,9,10,11,12].

Using next-generation sequencing (NGS) approaches, multiple insect infecting viruses have been characterized in other insects, like mosquitoes and sand-flies, such as those viruses belonging to the *Iflaviridae* family and the newly described *Negevirus* taxon [13,14]. The *Iflaviridae* family comprises positive-sense single-stranded RNA (+ssRNA) viruses in the order *Picornavirales* and contains a single genus, *Iflavirus*. The Iflavirus ssRNA genome encodes a single, large polyprotein, which is post-translationally processed into structural and non-structural viral proteins essential for its replication, packaging and transmission [13]. The single polyprotein is flanked by a 5′ untranslated region (5′ UTR), which includes an internal ribosome entry site (IRES) structure required for the cap-independent translation and a 3′ UTR with a poly(A) tail to terminate translation [15,16].

Viruses in the newly described *Negevirus* taxon are enveloped and have a +ssRNA genome of about 9 to 10 kb with a poly(A) tail at the 3′ end and encodes three major open reading frames (ORFs). The largest Negevirus ORF (ORF1) is approximately 7 kb and encodes the viral non-structural proteins: ribosomal RNA (rRNA) methyltransferase (MTase), viral MTase, helicase and the RNA-dependent RNA polymerase (RdRp). ORFs 2 and 3 are approximately 1.5 kb and 600 base pairs (bp), and they have been hypothesized to encode structural proteins, the glycoprotein and membrane protein, respectively [17]. Two major distinct clades or genera have been designated within the *Negevirus* taxon, the *Nelorpivirus* and the *Sandewavirus*, with the former being more closely related to the plant cileviruses and higreviruses [18]. However, further experimental studies are required to understand the mode of transmission, pathogenicity and replication of Negeviruses [19].

In tsetse flies, further investigations are required to provide crucial information on presence of persistent +ssRNA virus infections and how these might interact with the above-mentioned tsetse symbionts and GpSGHV. Here, we report the nucleotide sequences, genome organization and phylogenetic placement of two novel +ssRNA viruses belonging to *Iflaviridae* family and *Negevirus* genus, respectively, both isolated from *Glossina morsitans morsitans* reared at the Insect Pest Control Laboratory in Seibersdorf, Austria. The tissue tropism of these viruses was analyzed to provide insights into the possible transmission mode. RT-qPCR and Stellaris RNA *in situ* hybridization techniques were applied to detect the viruses in the various organs.

## 2. Materials and Methods

### 2.1. Sample Collection/Experimental Flies and Procedures

Tsetse flies (*G. m. morsitans*) used in this study were obtained from the colony maintained at the Joint FAO/IAEA Center, Insect Pest Control Laboratory (IPCL), Seibersdorf, Austria. The flies were maintained in an insectary at 23 ± 1 °C, 75–80% relative humidity, with a 12 h photo-phase and were fed defibrinated bovine blood for 10 to 15 min, three times per week using an in vitro membrane feeding system.

### 2.2. Discovery of RNA Virus Sequences and Genome Annotation

RNA-Seq data (SRR965341) derived from a pool of 30 salivary glands of trypanosome-infected *G. m. morsitans* and produced using the Illumina HiSeq 1000 was downloaded from the NCBI database. The assembly and mapping of these data were performed on CLC Genomic WorkBench (GWB) version 11.0.1 to generate contigs. The contigs that mapped to other related viruses were used to design the initial primers to detect these viruses in *G. m. morsitans* samples from IPCL. A series of forward and reverse primers were designed to join the non-overlapping contigs that mapped to the same virus family. The 5′ and 3′ ends of each virus were amplified by rapid amplification of cDNA ends (RACE) using the 5′/3′ RACE Kit, 2nd Generation (Roche Applied Science, Mannheim, Germany) in combination with virus-specific primers. Sequences and positions of the primers for the specific viral genomes used in this study are described in Table 1.

The assembled virus genome sequences were characterized based on their size, organization, and the encoded proteins. Briefly, the nucleotide sequences for both genomes were subjected to ORF finder (https://www.ncbi.nlm.nih.gov/orffinder/, accessed on 15 October 2018) to search for ORFs, while the amino acid sequences were used to scan for conserved functional domains and putative proteolytic processing sites using InterProScan (https://www.ebi.ac.uk/interpro/, accessed on 20 October 2018) and HMMER programs (https://www.ebi.ac.uk/Tools/hmmer/, accessed on 20 October 2018). The secondary structures of the 5′ UTR for both virus genomes were predicted using MFOLD program and ViennaRNA software package [20,21].

### 2.3. RNA Extraction and cDNA Synthesis

Total RNA was extracted from 10 individual whole bodies (5 males and 5 females) of 3–4 days old flies using TRIzol reagent (Invitrogen, Paisley, UK) according to the manufacturer’s instructions. The extracted RNA samples were treated with DNase 1 (Invitrogen, Paisley, UK) to remove any contaminating genomic DNA, and the RNA purity and concentration were determined using a Nanodrop ND-1000 spectrophotometer (Thermo Fisher Scientific, Wilmington, DE, USA). A total of 500 ng of RNA was reverse transcribed to complementary DNAs (cDNAs) using the SuperScript^®^ III Reverse Transcriptase kit (Invitrogen, Paisley, UK) and the oligo-dT primer included in the kit following the manufacturer’s instructions.

### 2.4. Virus Detection by RT-PCR

To detect the viruses in *G. m. morsitans* fly samples, one primer pair (Table 1) was designed from each of the contigs that mapped to the other viruses. The amplifications were carried out in 25 µL final reaction volume containing 2 mM of MgCl_2_, 0.2 mM of dNTPs mix, 0.2 μM of each primer, 1.0 U of Taq buffer, 0.25 μL of Go-Taq Polymerase and 1 µL of cDNA. The cycling conditions were 95 °C for 5 min, followed by 35 cycles of 95 °C for 45 s, 58 °C for 45 s and 72 °C for 45 s, then final extension of 72 °C for 10 min. The PCR products were analyzed by 1.5% agarose gel electrophoresis according to standard protocols.

### 2.5. Sequencing and Phylogenetic Analysis

All the PCR-amplified fragments for different contigs and the 5′/3′ RACE were purified following Illustra GFX PCR DNA and Gel Band Purification kit (GE Healthcare, Buckinghamshire, UK) and sequenced by Sanger sequencing technology. Multiple alignments of the identified sequences were performed in BioEdit [22] as well as on MEGA6 [23]. Phylogenetic analysis was performed with MEGA6 using the default settings for neighbor-joining method with bootstrap test of 1000 replicates.

### 2.6. Detection of Replicative RNA Strand by RT-PCR

To detect the replicative negative RNA strand of the identified viruses, cDNA was synthesized using a forward virus-specific primer (complementary to the negative RNA strand) from a DNase-treated total RNA. To confirm that the cDNA was generated from the virus negative strand, a PCR was performed using a primer pair located downstream of the forward virus-specific primer used at the reverse transcriptase (RT)-step. For each of the analyzed samples, a negative control without RT enzyme (NRT), a positive control (cDNA synthesized from the virus positive RNA strand) and no-template (RNase-free water as template) samples were included in this assay. The primers used are detailed in Table 1.

### 2.7. Tissue Distribution of Iflavirus and Negevirus Infection in Adult Tsetse Flies

To determine the tissues infected with GmmIV and GmmNegeV in adult tsetse flies *G. m. morsitans*, quantitative real time PCR (RT-qPCR) and the Stellaris RNA-Fluorescence *in situ* Hybridization (FISH) techniques were applied for detection and visualization of the viruses, respectively, in different tsetse isolated tissues. To isolate the tissues, 30-day-old flies were starved for 2 to 3 days then chilled at 4 °C for 15 min prior to dissection and were kept on ice during the dissection of different tissues. The tissues (salivary glands, hindgut, Malpighian tubules, midgut, foregut, ovaries, spermatheca, milk glands, testes, fat bodies and brains) were dissected in sterile phosphate-buffered saline (1X PBS) under a dissection microscope. Tissues for RT-qPCR analysis were collected in microtubes containing RNA later, while those for FISH were collected in fixating solution (3.7% paraformaldehyde, 1X PBS, 0.3% Triton-X 100; nuclease-free water) and were kept on ice during the dissection process. For the RT-qPCR analysis, the same tissues from 10 adult flies were pooled together and considered as one replicate. Male and female tissues were separated, with a total of three replicates for each sex.

#### 2.7.1. Relative Expression of Iflavirus and Negevirus in Adult Tsetse Tissues Using RT-qPCR

To detect and quantify GmmIV and GmmNegeV from the dissected tissues, total RNA was extracted and cDNA was synthesized as described in Section 2.3, followed by quantitative real time PCR (RT-qPCR). The RT-qPCR reactions were prepared in a 15 μL reaction volume containing 5 μL of (2.5 times diluted) cDNA template, 200 nM of each forward and reverse primer (Table 1), 1.5 µL ddH_2_O and 7.5 μL iQTM SYBER Green Supermix (Bio-Rad laboratories, Hercules, CA, USA). Three technical replicates per virus target and housekeeping (*β-tubulin*) gene were performed. The PCR was performed in a CFX96 PCR machine (Bio-rad laboratories, Hercules, CA, USA), with an initial denaturation step at 95 °C for 2 min, followed by 39 cycles of 95 °C for 5 s, 62 °C for 30 s, ended by a fluorescence read and 95 °C for 5 s, followed by a melting curve from 65 to 95 °C. The real-time data were analyzed using the 2^(−ΔΔct)^ method using the CFX Maestro software version 2 (Bio-Rad).

#### 2.7.2. Detection of Iflavirus and Negevirus in Adult Tsetse Tissues Using FISH

To visualize the two viruses in adult tsetse flies using FISH technique, custom probe sets for GmmIV and GmmNegeV were designed using the Stellaris^®^ FISH probe designer (www.biosearchtech.com/support/tools/design-software/stellaris-probe-designer, accessed on 27 May 2020) according to a protocol previously described by Schneider et al. [24]. The endosymbiont bacteria *Wolbachia* were also analyzed using W1 and W2 probes targeting 16S rRNA [25] of the bacterium (Table S1) as a positive control for the technique. The isolated tissues were first fixed for 20 min at room temperature under constant gentle shaking, then washed three times for 10 min each in PBS-T (1X PBS with 0.3% Triton-X 100). The tissues were rinsed one time with 70% ethanol and kept in fresh 70% ethanol overnight at 4 °C at constant agitation. The following day, the tissues were washed briefly in sterile 1X PBS and rehydrated for 5 min in prewarmed washing buffer (4X saline sodium citrate [SSC], 20% deionized formamide, DEPC treated water) at room temperature. The tissues were then hybridized overnight at 37 °C in a dark and humid hybridization chamber in 50 μL hybridization mix (0.1% dextrane sulfate, 4X SSC, 20% deionized formamide, DEPC treated water), containing 0.3 pmol of each Stellaris^®^ RNA-probe and 3 pmol of W1 and W2 *Wolbachia* probes. Following hybridization, the tissues were briefly washed 3 times with preheated washing buffer at room temperature followed by one wash for 30 min at 37 °C in the dark hybridization chamber. Tissues were then incubated with Alexa Fluor^®^ 488 phalloidin (Invitrogen, Paisley, UK) staining F-actin (1:100 in 1X PBS) for 1 h, shaking at room temperature in the dark. Alexa Fluor^®^ 488 phalloidin was then replaced by 1X PBS, and the tissues were mounted in Roti-Mount© FluorCare (Carl Roth, Karlsruhe, Germany), containing DAPI (4′,6-diamidino-2-phenylindole) on sterile slides. Visualization of the hybridized tissues was performed on either a Nikon A1 or an Olympus FluoView 3000 confocal microscope. *G. pallidipes* was used as a negative control because the two viruses investigated here have remained undetectable in this species by RT-PCR.

### 2.8. Statistical Analyses

RT-qPCR data were analyzed with the general linear model (GLM) on R v 4.0.5 [26] using RStudio V 1.4.1106 [27,28] with packages ggplot2 v3.3.2.1 [29], lattice v0.20-41 [30], car [31], ggthems [32] and MASS v7.3-51.6 [33]. The target expression levels were normalized to *β-tubulin* expression.

## 3. Results

### 3.1. Discovery of the RNA Viruses in G. m. morsitans

**Figure 1:**
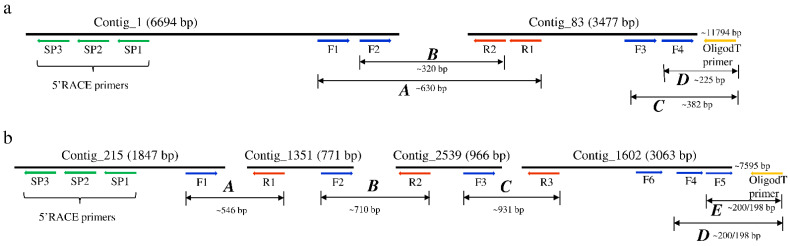
Schematic representation of the assembled contigs mapping to (**a**) Glossina morsitans morsitans iflavirus and (**b**) Glossina morsitans morsitans negevirus genomes. Positions of the primers used to join the contigs and the 3′ and 5′ untranslated regions (UTR) to obtain the complete viral genomes are shown.

The sequences used for the discovery of the RNA viruses in tsetse originated from the RNA-seq data of both trypanosome infected and non-infected *G. m. morsitans* flies. The assembly of the RNA-seq data in CLC GWB generated 6843 contigs containing >500 reads. Six contigs mapped to two positive single-stranded RNA (+ssRNA) viruses. Using BLASTx, two non-overlapping contigs (#1 and 83) mapped to a non-annotated Kinkell iflavirus sequence (AMO03216.1) with 43 and 45% sequence identity, respectively, while four other non-overlapping contigs (#215, 1251, 2539 and 1602) mapped to Negeviruses, a newly identified insect-infecting virus group. Contigs #1351 and 1602 showed low levels (23–61%) of amino-acid identity with Dezidougou virus (JQ675604.1) and Tanay virus (KF425262.1), and contigs #215 and 2539 showed 47% and 36% of amino-acid identity with Tanay virus (KF425262.1). To confirm the presence of the two viruses, the initial RT-PCR was performed on the *G. m. morsitans* samples using primer sets located in each of the identified contigs and sequenced (Figure 1). RT-PCR using a series of forward and reverse primers successfully joined the non-overlapping contigs of both viruses, whereas the 5′ and 3′ untranslated regions (UTR) were obtained using 5′ and 3′ RACE technique (Figure 1). Therefore, the two viruses are proposed to be named as Glossina morsitans morsitans iflavirus (GmmIV) and Glossina morsitans morsitans negevirus (GmmNegeV). The genome sequences of GmmIV and GmmNegeV were submitted to GenBank receiving the accession numbers OL353434 and OL353435, respectively.

### 3.2. Viral RNA Genome Analysis and Organization

**Figure 2:**
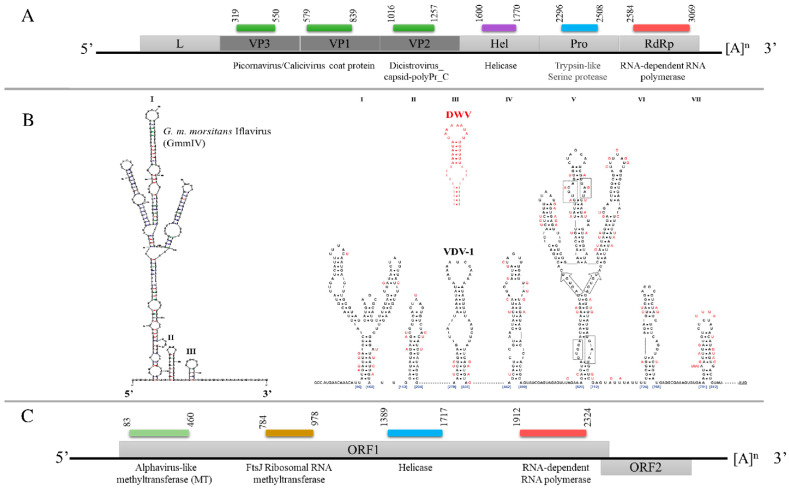
Schematic representation of the genome organization of (**A**) GmmIV, indicating the structural proteins (dark gray) and the non-structural proteins (light gray). (**B**) Predicted 5′ UTR secondary structures of GmmIV in comparison to well-studied 5′ UTR hairpins of Varroa destructor virus 1 (VDV-1) in black and deformed wing virus (DWV) in red [16]. (**C**) Genome organization of GmmNegeV showing the predicted open reading frames. The conserved functional domains and their positions on each viral genome, the 5′ UTR, 3′ UTR and the poly(A) tails, are also shown. L, leader protein; VP, virion protein; Hel, helicase; Pro, trypsin-like serine protease; RdRp, RNA-dependent RNA polymerase.

The assembled GmmIV genome sequence contains 9685 nucleotides (nts) with a single, large ORF (9222 nts) encoding a polyprotein that is cleaved into both structural proteins (capsid proteins located at the N terminal) and non-structural proteins. The viral genome contains a single ORF, which is flanked by a 5′ UTR (300 nts) and a 3′ UTR (163 nts) that ends in a poly(A) tail (Figure 2A). The encoded polyprotein contains several conserved domains: two picornavirus-like capsid protein domains, which correspond to VP3 and VP1, and a cricket paralysis virus capsid protein-like domain, corresponding to VP2. The conserved domains identified for the non-structural proteins were an RNA helicase and RdRp. The analysis of the 300 nts of the 5′ UTR demonstrated three stable secondary structures. These included one hairpin (I) that formed branched structures, which are similar to a hairpin (V) of the IRES structures described for Deformed wing virus (DWV) and Varroa destructor virus 1 (VDV-1) [16] in terms of shape and distance from the ORF (Figure 2B) which may have a similar function in translation initiation.

The GmmNegeV genome isolated from *G. m. morsitans* is composed of 8140 nts encoding two overlapping ORFs flanked by 5′ UTR and 3′ UTR. The GmmNegeV ORF1 (7035 nts) encodes a polyprotein containing four typical non-structural protein domains: a methyltransferase (MT) domain, a ribosomal RNA methyltransferase domain, a viral RNA helicase domain and an RdRp domain (Figure 2C). The second ORF (ORF2) is located downstream in a +1 reading frame from ORF1, and partly overlaps. However, it makes use of another reading frame and encodes three glycosylated proteins (_23_NITT_26_, _149_NSSG_152_ and _245_NYTD_248_) that are associated with the viral envelope.

### 3.3. Phylogenetic Analysis

**Figure 3:**
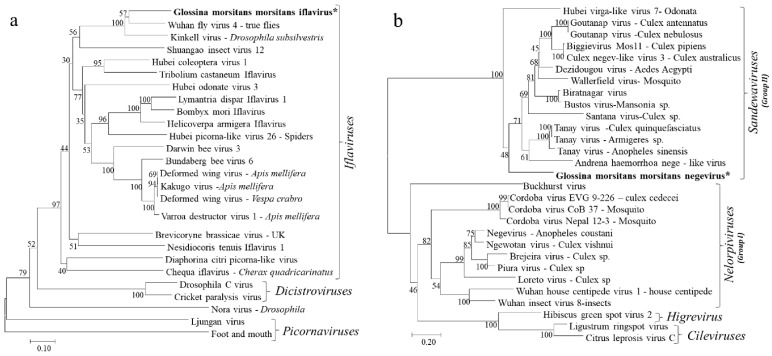
Phylogenetic analysis based on the aligned RdRp amino acid sequences of (**a**) Iflaviruses and other viruses in the order *Picornavirales* and (**b**) Negeviruses. The phylogenetic trees were constructed using the neighbor-joining method with bootstrap test of 1000 replicates. The newly identified Iflavirus and Negevirus in *G. m. morsitans* are indicated by the asterisk (*) and in bold.

Due its highly conserved nature among insect RNA viruses, the deduced amino acid sequences for the RdRp proteins were used in phylogenetic analyses of the identified GmmIV and 25 other viruses in the order *Picornavirales* in GenBank. The RdRp segregated the viruses into taxonomic groups (*Picornaviridae*, *Iflaviridae* and *Dicistroviridae* families) within the order *Picornavirales* and clustered GmmIV with viruses belonging to the *Iflaviridae* family such as Wuhan fly virus 4 (Figure 3a). In addition, the phylogenetic analysis of amino acid sequences of the GmmNegeV RdRp with that of other Negeviruses clustered GmmNegeV with group II sandewaviruses, as reported in other insects such as Goutanap virus, Dezidougou virus and Tanay virus (Figure 3b).

**Figure 4:**
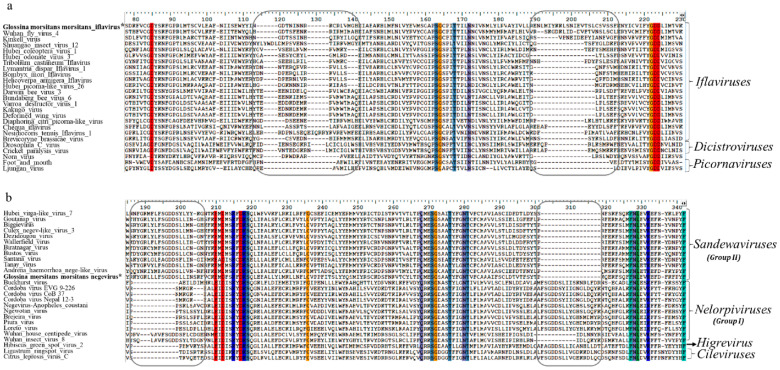
Alignment of RNA-dependent RNA polymerase (RdRp) coding amino acids of (**a**) Iflaviruses and (**b**) Negeviruses displaying the conserved deletions and insertions (in blocks) in the RdRp. Amino acids conserved in all sequences are highlighted in different colours. The newly identified Iflavirus and Negevirus in G. m. morsitans are indicated by the asterisk (*) and in bold.

The phylogenetic placement of both GmmIV and GmmNegeV in their respective virus taxon was also supported by motif patterns observed in the RdRp amino acids alignments (Figure 4). Conserved deletions and insertions were also observed in GmmIV and GmmNegeV RdRp amino acids sequence alignments, which were like other viruses belonging to the *Iflaviridae* family and *Negevirus* group II sandewaviruses, respectively (Figure 4). A comparison of RdRp amino acid identity showed that GmmIV shared 64.4% amino acid identity with Wuhan fly virus *4*, while GmmNegeV shared 55.4%, 58.6% and 56.7% with the Goutanap virus, Dezidougou virus and Tanay virus, respectively.

### 3.4. Detection of Replicative RNA Forms of Iflavirus and Negevirus in G. m. morsitans

**Figure 5:**
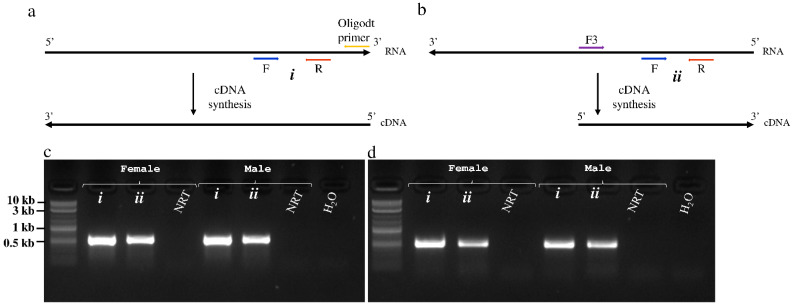
Detection of positive and negative RNA strands of Glossina morsitans morsitans iflavirus (GmmIV) and negevirus (GmmNegeV). (**a**) cDNA synthesis approach from the positive RNA strand using oligo-dT primer. (**b**) cDNA synthesis from the replicative negative RNA strand using a virus-specific forward primer. The gel image shows the amplified fragments of (**c**) GmmIV and (**d**) GmmNegeV using specific primer pairs for each virus in addition to cDNA originating from the positive RNA strand (i) or negative RNA strand (ii) as the template from both male (♂) and female (♀) flies. NRT, non-RT enzyme control.

During replication of +ssRNA viruses, a complementary negative RNA strand is normally transcribed from the positive RNA genome, which serves as the template for multiplication of the viral genome to form new virus progeny. To provide evidence that both GmmIV and GmmNegeV replicate in tsetse flies, a strand-specific RT-PCR was performed using cDNA transcribed from either positive and negative RNA strands of the two viruses (Figure 5a,b). A primer pair located downstream of the RT-step forward primer which is complementary to the virus negative RNA strand was used for the PCR. A PCR product of 575 bp was amplified with GmmIV primers in combination with GmmIV templates (Figure 5c), and a 540 bp product with GmmNegeV primers in combination with GmmNegeV templates (Figure 5d). Both GmmIV and GmmNegeV replicate in *G. m. morsitans* as seen by RT-PCR amplification of the specific negative strands (Figure 5c,d lanes ii). In addition, both female and male *G. m. morsitans* were infected with both viruses as confirmed by the detection of GmmIV and GmmNegeV positive RNA strand genomes (Figure 5c,d lanes i). No PCR products were observed when the RT step was omitted (NRT) or when RNase free water (H_2_O) was used as template. The RT-PCR products from both positive and negative RNA templates were sequenced to confirm the sequences of both viruses.

### 3.5. Tissue Distribution of Iflavirus and Negevirus Infection

#### 3.5.1. Assessment of Iflavirus and Negevirus Density in Different Tsetse Tissues by RT-qPCR

**Figure 6:**
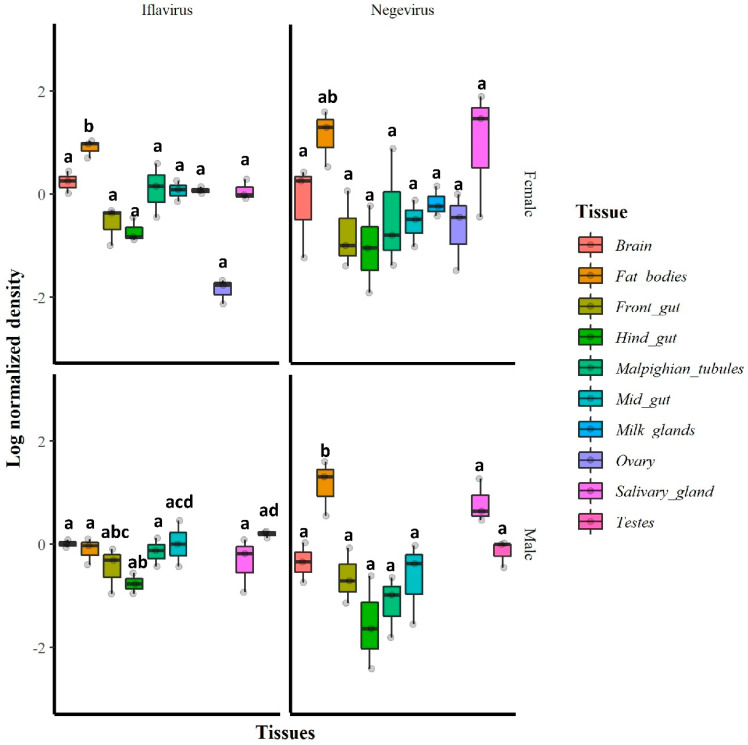
Relative densities (log_10_) of GmmIV and GmmNegeV in *G. m. morsitans* tissues: RT-qPCR was conducted on total RNA extracted from 30-day-old female (**top**) and male fly tissues (**bottom**) and normalized to *β-tubulin* gene expression. Bars marked with the same lower-case letter do not differ significantly at the 0.05 level.

The RT-qPCR results indicated that in general, all tested tissues were infected with GmmIV and GmmNegeV. The densities of both GmmIV and GmmNegeV varied significantly in different tissues regardless of the fly sex (GmmIV: X^2^ = 28.65, df = 9, *p* = 0.0007; GmmNegeV: X^2^ = 29.21, df = 9, *p* < 0.01). In particular, GmmIV density varied in male fly tissues (X^2^ = 14.19, df = 7, *p* = 0.0479) with the highest density in testes and midguts and the lowest detected in the hind gut, while in female fly tissues (X^2^ = 102.5, df = 8, *p* < 0.001), the highest GmmIV density was observed in fat bodies and the lowest in the ovaries (Figure 6). The GmmNegeV density varied in both male (X^2^ = 23.392, df = 7, *p* < 0.001) and female (X^2^ = 18.43, df = 8, *p* = 0.018) tissues, with the highest density in the fat bodies and salivary glands and the lowest in hind gut and Malpighian tubes in both sexes (Figure 6).

#### 3.5.2. Stellaris FISH

**Figure 7:**
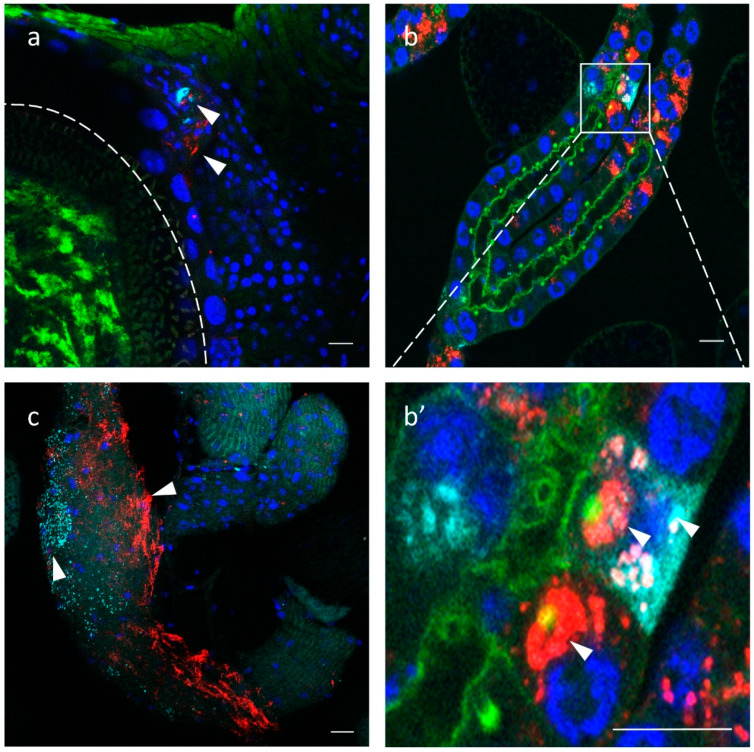
Iflavirus and Negevirus double staining in the gonads and milk glands of 30-day-old *G. m. morsitans* female and male flies: GmmIV—red, GmmNegeV—cyan, *Wolbachia* and Phalloidin (F-actin) —green, nucleus—blue. (**a**) Ovaries, oocyte border is depicted with the dashed line, (**b**) milk glands, (**c**) testes, (**b’**) is a higher magnification of (**b**) milk glands. Scale bar: 20 μm.

To localize the distribution of GmmIV and GmmNegeV in different *G. m. morsitans* tissues, we applied the Stellaris RNA Fluorescence *in situ* hybridization technique. The reproductive organs of *G. m. morsitans* in both females and males showed a presence of GmmIV and GmmNegeV (Figure 7a,c). In the ovaries, a double infection of GmmIV and GmmNegeV was observed in the oviduct together with high *Wolbachia* infection of the oocyte (Figure 7a). Only a few cells of the ovaries were infected with the viruses, which coincides with the overall low virus density detected with RT-qPCR (Figure 6). The testes exhibited a much stronger infection with GmmIV and GmmNegeV (Figure 7c) with the former having the highest abundance. This finding supports the results of the RT-qPCR data that demonstrated high density of GmmNegeV and a significantly higher presence of GmmIV compared to the other organs (Figure 6).

The milk glands showed high prevalence of both GmmIV and GmmNegeV in the secretory cells (Figure 7b). Some cells were single-infected with either GmmIV or GmmNegeV and others were double-infected, with each virus mostly confined in a separate cell niche (Figure 7b’).

**Figure 8:**
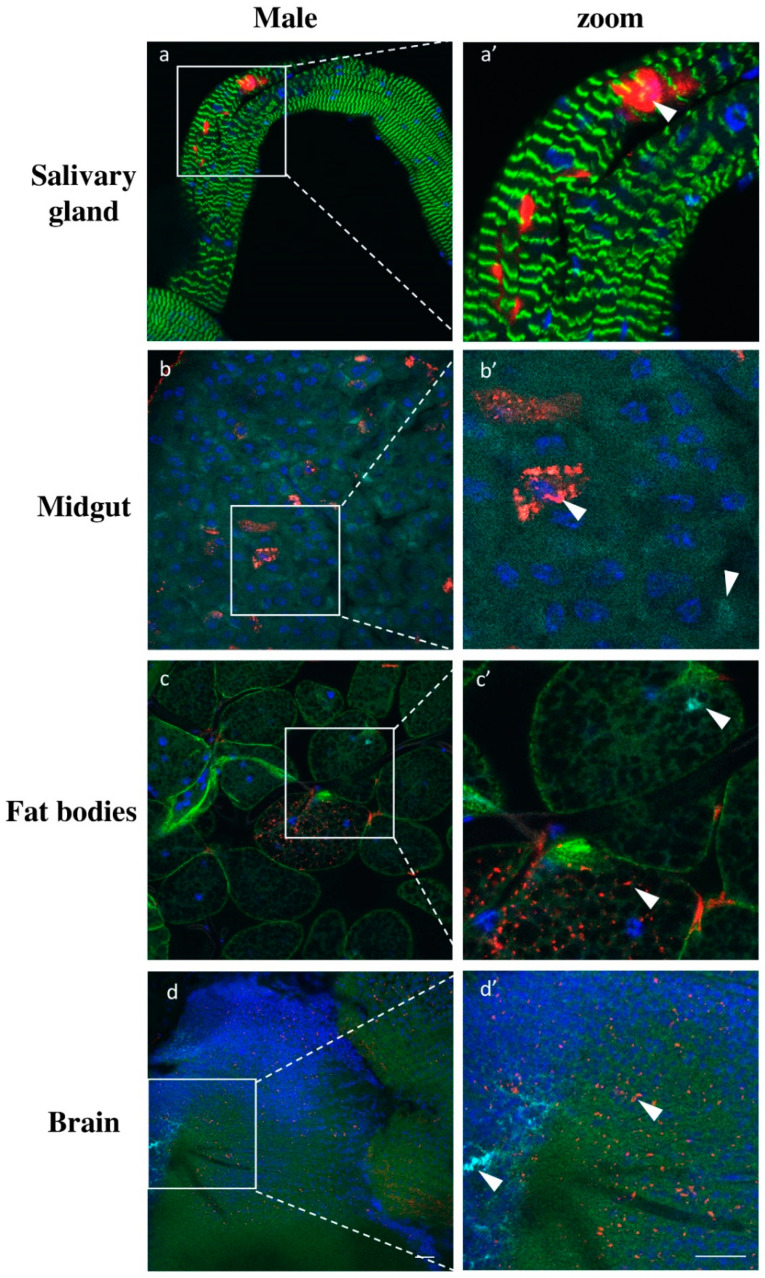
Double staining GmmIV and GmmNegeV in different tissues of 30-day-old *G. m. morsitans* male flies: GmmIV—red, GmmNegeV—cyan, actin—green, nucleus—blue. (**a’**–**d’**) are higher magnification of (**a**–**d**) images. Scale bar: 20 μm.

Additionally, we analyzed the viruses’ infection in other somatic tissues besides the milk glands. For all tissues analyzed, we did not detect any sex specific differences in infection patterns. Salivary glands exhibited only low GmmIV infection found exclusively in epithelial cells and not in the lumen of the organ (Figure 8a). The absence of GmmNegeV infection in salivary glands contradicts the RT-qPCR results where we observed a high density of the virus, particularly in females (Figure 6 and Figure S1). In the midgut, we could mainly observe the presence of GmmIV and a lower prevalence of GmmNegeV. The viruses were mainly restricted to a few cells in the tissue with most of the cells being free of infection (Figure 8b). A similar infection pattern was observed for the fat body where viruses inhabit separate adipocytes (Figure 8c). In brains, both viruses were stochastically distributed all over the organ with GmmNegeV forming clusters and rarely sharing the same niche with GmmIV, which had a more systemic localization (Figure 8d). The negative control tissues from *G. pallidipes* did not show any positive fluorescence signal for either GmmIV or GmmNegeV, which excludes any possible cross hybridization (Figure S2).

In summary, we observed both viruses in reproductive tissues of the *G. m. morsitans* flies with much higher titer in the testes. Most of the somatic tissues were double-infected with both viruses, often occupying separate cells and rarely sharing the same environment.

## 4. Discussion

In this study, we report the discovery of two novel viruses from *G. m. morsitans* which belong to the *Iflavirus* and *Negevirus* taxa [13,14], respectively. The newly identified Iflavirus is proposed to be named as Glossina morsitans morsitans iflavirus (GmmIV). The GmmIV has an ssRNA genome of 9685 nts in length, including the poly(A) tail, and contains a single, large ORF encoding a 3074 aa polyprotein which contains both structural and non-structural proteins essential for virus replication and polyprotein processing. Based on the GmmIV genome organization and amino acid sequence similarity with other viruses belonging to the genus *Iflavirus*, we propose GmmIV as a novel member of this genus [13]. The analysis of the GmmIV 5′ UTR showed it to be longer (300 nts) than that of Wuhan fly virus 4 strain (101 nts) [34], which was phylogenetically grouped together but distinctly shorter compared to more distantly related Iflaviruses such as DWV (1144 nts) or VDV-1 (1108 nts), which are distantly related to GmmIV [16]. However, these length differences at 5′ UTR could be due to incomplete sequencing of the 5′ ends for some of the reported viruses, or they may represent differences in their functional significance. Compared to the viruses belonging to *Dicistroviridae* or *Picornaviridae* families, *Iflavirus* genomes lack a 5′ cap structure for the initiation of protein synthesis; instead, they use an IRES for translation initiation [35]. So far, functional IRES elements have only been predicted for Iflaviruses with long 5′ UTR sequences such as DWV, VDV-1 and Perina nuda virus (PnV) [16,36]. However, due to the similarities found of the GmmIV 5′ UTR secondary structures to the above-mentioned Iflaviruses, it is likely that it may contain functional IRES elements, whose interactions with host translation initiation factors require further studies.

We also showed that tsetse flies are infected by a new Negevirus, here proposed to be named as Glossina morsitans morsitans negevirus (GmmNegeV). To date, Negeviruses have only been associated with mosquitoes and sandflies isolated in a number of geographic locations, including Europe, United States, Indonesia and Africa [14,19]. Like other characterized Negeviruses, the newly identified GmmNegeV has a (+) ssRNA genome of about 8500 nts in length flanked by 5′- and 3′ UTRs. Based on the genome organization, the GmmNegeV genome contained only two overlapping ORFs as opposed to most of the Negevirus genomes that encode three ORFs [17]. A genome comparative study of insect-specific negeviruses reported that this group of viruses may encode between one to three ORFs in their genomes, which may be overlapping or separated by intergenic regions, but they all possess at least one large ORF which encodes the four conserved domains of the virus non-structural proteins [17]. Reported Negeviruses encoding less than three ORFs in their genomes are the Cordoba virus strains (encoding one ORF) and the Buckhurst virus (encoding two ORFs).

Phylogenetic analysis of the insect-specific negeviruses classify them into two clades; nelorpiviruses and sandewaviruses, which suggests two separate origins of the viruses belonging to these two groups, with nelorpiviruses being more closely related to plant viruses (cileviruses and higreviruses) [17]. In this study, based on the amino acid sequences of the Negeviruses ORF1, GmmNegeV not only showed a high degree of similarity (54.5–58.6%) to the sandewaviruses, but was also phylogenetically placed in this group based on the RdRp aa alignment. Other examples of viruses belonging to the *Sandewavirus* group are Tanay virus, Santana virus, Bustos virus, Dezidougou virus and Goutanap virus while those belonging to the nelorpiviruses are Negewotan virus, Brejeira virus, Piura virus and Loreto virus [18,19]. Due to this close relationship of insects infecting negeviruses to plant viruses (cilevirus, higrevirus), they are therefore thought to be acquired from plants by insects feeding on plant juices such as nectar [37,38]. This is also likely for the strictly hematophagous tsetse flies that have recently been reported to feed on other sources of food other than blood in the field [39].

Our study has provided compelling evidence that GmmIV and GmmNegeV replicate in *G. m. morsitans*, as clearly confirmed by detection of the replicative negative strand RNA of GmmIV and GmmNegeV. Furthermore, we investigated tissue tropism of these viruses in *G. m. morsitans* as a model. Until now, Iflaviruses and Negeviruses were only known to be present in mosquitoes, sandflies and beet armyworm [14,40]. Nothing was known about the presence of these viruses in teste flies, nor about the impact of their infections on the tsetse colony performance. We hypothesize that some of the tsetse species may carry Iflavirus and Negevirus in a covert asymptomatic state and that the transmission of these viruses must occur vertically from parent to offspring. In line with this hypothesis, it is also likely that if the virus titers are detectable in the salivary glands by RT-PCR or RNA-FISH, horizontal transmission between adult flies would be also possible. We demonstrated that both GmmIV and GmmNegeV can be found in the reproductive organs, as well as in milk and salivary glands, suggesting a horizontal transmission of the virus during feeding and/or a vertical viral transmission from parents to offspring.

A vertical transmission has already been demonstrated in the case of GpSGHV in *G. pallidipes* [7], which might be associated with the presence of the tsetse symbiont *Sodalis* [41]. In our study, we confirmed the presence of tsetse symbionts in the milk glands, but further investigations are needed to understand their role in the transmission of GmmIV and GmmNegeV. Horizontal viral transmission between adult tsetse flies has also been demonstrated previously by Kariithi et al. [4], who showed that GpSGHV horizontal transmission occurs when infected flies feed on the same blood pool. The flies inject the virus in the blood via their salivary glands [4]. In our study, the salivary glands did not show high densities of GmmIV and GmmNegeV using the *in situ* technique, but both viruses were detected by RT-qPCR. As such, the viruses may only be present in the saliva itself but remain undetectable in the epithelial cells by the *in situ* technique. Another reason for the low presence of the viruses in the salivary glands might be that during low rearing densities of tsetse flies, vertical transmission might be favored. However, further experiments are needed to understand the impact of different tsetse rearing densities on these viruses’ horizontal transmission. In addition, all the experiments in this study were performed on colony flies and therefore, data on the possible presence of these two newly described viruses in natural glossinidae fly populations would be crucial as the virus dynamics in the wild may be different as the environment changes drastically.

In tsetse rearing facilities, it is unclear if the presence of GmmIV and GmmNegeV has a beneficial or a detrimental effect on the flies. As reported for most Iflaviruses and all Negeviruses [42], none of the GmmIV and GmmNegeV infected *G. m. morsitans* flies analyzed in this study showed any overt virus infections. Notably, *G. pallidipes* was the only species at the IPCL that both GmmIV and GmmNegeV were undetectable, and it is the most susceptible species for GpSGHV overt infections [43]. This may imply that GmmIV and GmmNegeV could be beneficial to the tsetse immune system, thereby protecting some tsetse species from overt virus infections such as GpSGHV. This has been demonstrated to be a result of competition for host intracellular molecules [44]. It is tempting to speculate that these small RNA viruses permanently alert the RNAi defense response. Alternatively, it may be possible that GmmIV and GmmNegeV are opportunistic pathogens and may become virulent under certain environmental factors. These questions will be addressed in upcoming experiments.

## 5. Conclusions

In conclusion, this study expands the list of viruses infecting tsetse flies in addition to GpSGHV as well the symbiotic bacteria harbored by tsetse flies. However, the host range and better understanding of these virus−symbionts−host interactions as well as their effects on tsetse vector competence to trypanosome require further investigation.

## Figures and Tables

**Table 1:** Primers used for RT-PCR of GmmIV and GmmNegeV are given in 5′-3′ orientation.

Primers for GmmIV 5′ RACE	
IflaTse_SP3	ATAGATGCAGGATGGTTAGGTTGCT
IflaTse_SP2	GAGCCTGATGGATGTTGTGTTTGT
IflaTse_SP1	ACACCATACTTACACACAGCCATTC
Primers for GmmIV detection	
Iflav-tseCont1-1R	AAATGGCTACGCGATGTAGAATGG
Iflav-tseCont1-1F	TTTGCCTTTGTCCTTTAGATGTGCT
IflaTse_C1-F1	TGTTGGTGCTAGATTTAAGGAAAGGT
IflaTse_C1-F2	TTGAATTAGTTAAGCGATCTAGCCA
IflaTse_C83-R1	TCGGACATAGAATCAACAACAATACCA
IflaTse_C83-R2	AAGAACACTTCAATCTCTCTGCCAA
Primers to join the GmmIV contigs	
Iflav-tseCont83-1F	ATAGCCCCTAAAACAATAGCCCAAA
Iflav-tseCont83-1R	CCACACATTCCTCTACCATTTACTT
Iflav-tseCont83-2F	CAGGTATGGTTAGTGGTGAGAGAGG
Iflav-tseCont83-2R	GGACGAACAGAGGAAAACGGAAAAC
Primers for GmmIV 3′ RACE	
IflaTse_C83-F3-a	GCAATGGATAAGCGTGCAATAGAAG
IflaTse_C83-F3-b	TTTGGCGTAAAGAACGATTGGTG
Primers for GmmNegeV 5′ RACE	
NegeTse_SP3	TATGTAGCAATTTCGTTGAGAG
NegeTse_SP2	TTTACAGCATCAGCAGAATCCA
NegeTse_SP1	TGGAACGACAAGACGAATAGG
Primers for GmmNegeV detection	
NegeTse_C215-1F	TGTCTTGGTTTAGGAGTTTATTCGATGG
Negv-tseCont1351-1F	CCATTGTACTGAATTGCGTCCTAAGT
Primers to join GmmNegeV contigs	
NegeTse_C1351-1R	GTACGGATGAATCGCAAATAAATGA
Negv-tseCont1351-1R	CATAACGGCAGCGTCACTCATAAC
NegeTse_C1351-1F	CGGTAACGCTGTTGTTAAATCTT
NegeTse_C2539-1R	TTCATGTCAGCAACTCTAACAAATC
NegeTse_C2539-1F	CTTGTGACGTGGTCGCTGCTTT
NegeTse_C1602-1R	ACATACGCCTGTTGCGGATA
Negv-tseCont1602-1F	CTTCGTGTCCTAATGTTCGTTTTGT
Negv-tseCont1602-1R	GTTTTCCGTATTTTCTGTAAGCGTG
NegeTse_C1602-3F-a	AGCAAGGTGGATGGGTATATCTTGT
NegeTse_C1602-3F-b	TGATAAAGAACCTGTGTATGTTCCC
NegeTse_C1602-2R	TCTAAAGAAGGAAAGTCAGGGTTAC
Primers for GmmNegeV 3′ RACE	
NegeTse_C1602-2F-a	TATCCGAAGGTTATGGTTATGGTT
NegeTse_C1602-2F-b	TTTCCTCCTTCGTCTTATGTGA
NegeTse_C1602-3R	TAGTCACATAAGACGAAGGAGGA
	Primers for GmmIV and GmmNegeV RT-qPCR
Ifla_qPCR2_7848F	AGAAATTGAAGGACAGATGTTTGGT
Ifla_qPCR2_7947R	ACCTAAGAAATTACCAGTACCCTCC
Nege_qPCR1-2411F	CAACATAGACTTGAACCAGAGCA
Nege_qPCR1-2529R	GAAACATCAAACACACTCCCATTAG
Tsetse-tubulinF	GATGGTCAAGTGCGATCCT
Tsetse-tubulinR	TGAGAACTCGCCTTCTTCC

## Data Availability

The materials described in the paper, including all relevant raw data, are available at this link https://dataverse.harvard.edu/dataset.xhtml?persistentId=doi:10.7910/DVN/CNFRSB (accessed on 9 December 2021).

## Supplementary Materials

The following are available online at https://www.mdpi.com/article/10.3390/v13122472/s1, Figure S1: Double staining GmmIV and GmmNegeV in different tissues of 30-day-old *G. m. morsitans* female flies; Figure S2: Negative control of *G. pallidipes* FISH with stellaris probes for GmmIV and GmmNegeV demonstrating the absence of Iflavirus and Negevirus infection; Table S1: Stellaris RNA-FISH probes for GmmIV and GmmNegeV.
