# Supplementary material for: Characterization and Tissue Tropism of Newly Identified Iflavirus and Negeviruses in Glossina morsitans morsitans Tsetse Flies
Source: Viruses. 2021 Dec 10;13(12):2472. doi: 10.3390/v13122472 (PMC8704047; doi:10.3390/v13122472)
Supplement: Supplementary file 1 [file viruses-13-02472-s001.zip › viruses-1486185-supplementary material-1.pdf]

Supplementary materials

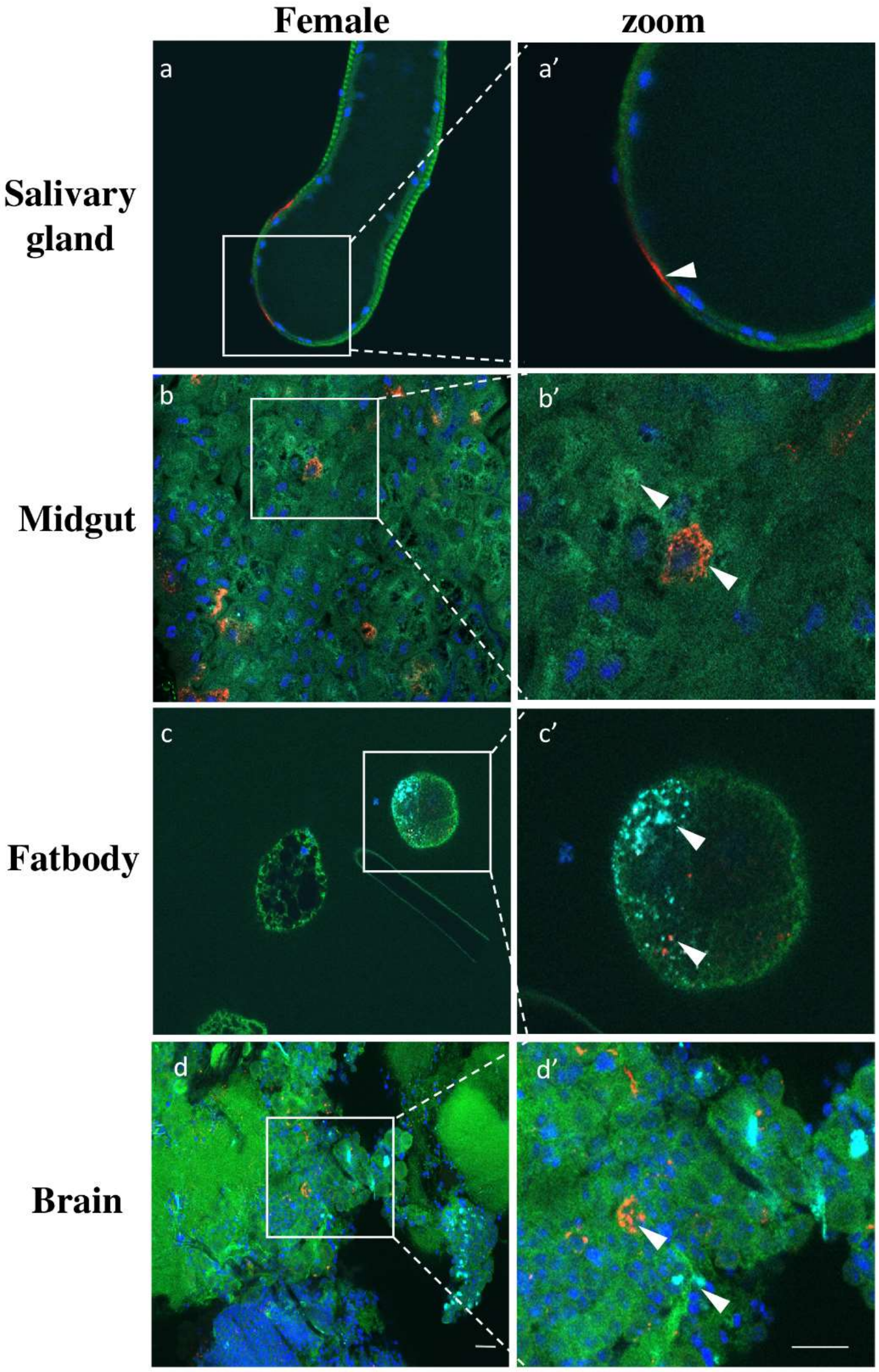

Figure S1. Double staining GmmIV and GmmNegeV in different tissues of 30-day old *G. m. morsitans* female flies: GmmIV - red, GmmNegeV - Cyan, Actin - green, Nucleus - blue. Scale bar: 20 μm.

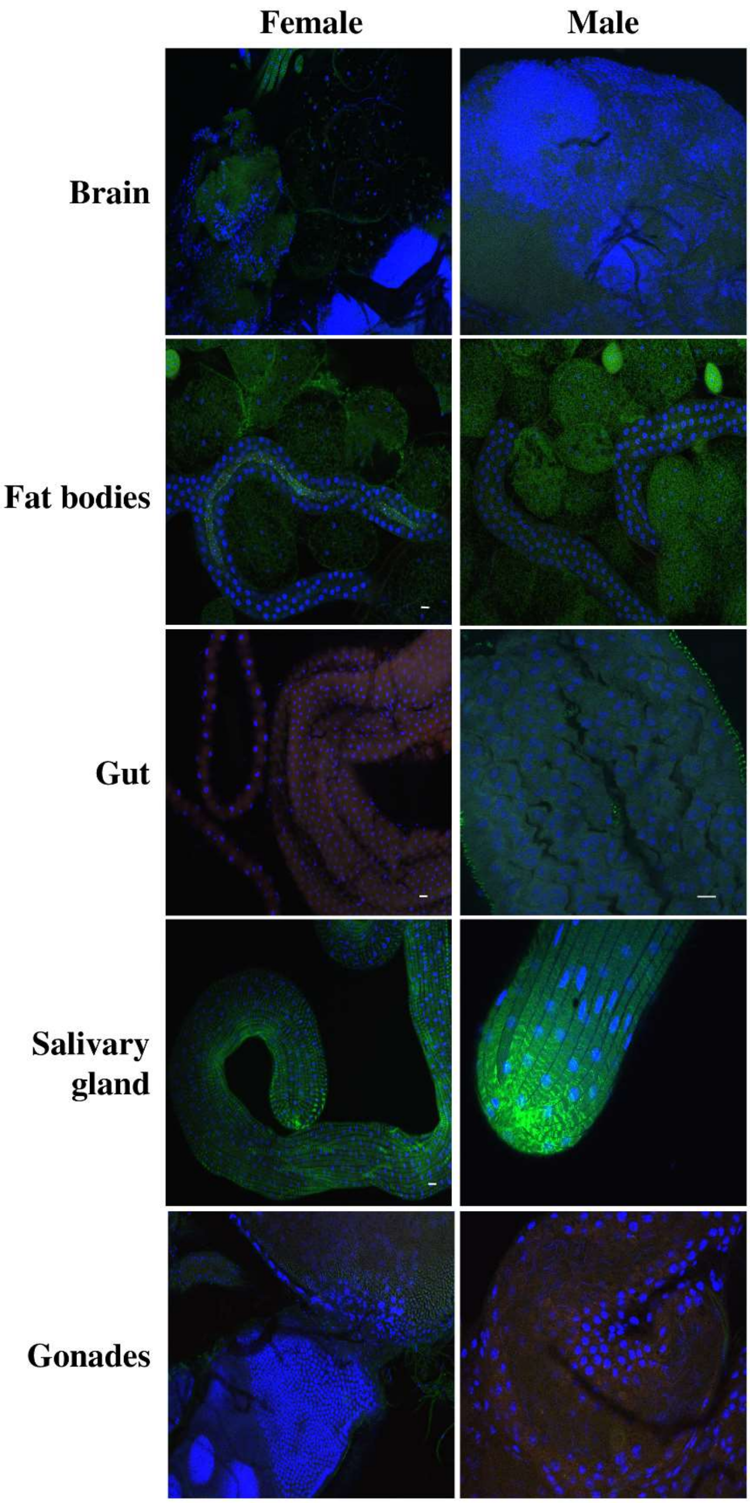

Figure S2: Negative control of *G. pallidipes* FISH with stellaris probes for GmmIV or GmmNegeV demonstrating the absence of Iflavirus and Negevirus infection. Magnification 20x and 40x for females and males respectively. Scale bar: 20 µm. blue: Nucleus (Dapi staining (4′,6-diamidino-2-phenylindole)), green: F-Actin staining (488 phalloidin), red: Iflavirus, red: Negevirus.
(females: GmmNegeV staining, males: brain, fatbodies, gut, saliary glands: GmmNegeV, Gonads: GmmIV).

File S1: Statistical analysis of the RT-qPCR data. (Rmarkdown attached)

Table S1: Stellaris RNA-FISH probes for GmmIV and GmmNegeV.

	**Probes for *Gmm* Iflavirus with Cal Fluor 610**		
**Number**	**Probe (5′→3′)**	**Probe position**	**% GC content**
1	TTAGCAGACTGACGACGGAG	508	55.00%
2	TTAATTGAGCGAGGCTCACG	430	50.00%
3	CGTAAGTACTACAGGTCCAG	1289	50.00%
4	ACCAATCTGGAAGGGATTCG	1595	50.00%
5	CAGCAGGTGAAGGTGAATCT	3204	50.00%
6	ATTTGAGGCGACAGACGTTG	3758	50.00%
7	CCCATAACGGTCACACAATA	109	45.00%
8	CGGGACATGATCACGTTTTT	385	45.00%
9	GAGCCGGTGTTAAAACTTCT	483	45.00%
10	AACACGACCTGGACTATCTT	677	45.00%
11	ACCCGAGATCTTGCTTAAAC	1149	45.00%
12	AGTACTACATCAGCAGCATC	1723	45.00%
13	GGCATCTAAGTGCACCTAAA	1815	45.00%
14	GGTGGTAATACAGCCATTTG	2353	45.00%
15	GCGAACCACGATTATATGCA	2397	45.00%
16	GGATTATACGGACGCTCATT	2584	45.00%
17	TATGAAGCTGCTGTCTCTTG	3136	45.00%
18	ACCTTGAGATACAACGGGTT	4199	45.00%
19	ACACCATCTTGAGGGATATC	4318	45.00%
20	GGTAGCCATGATAGAACGTT	4646	45.00%
21	CCTAAACCAAGCTATAGGGT	4859	45.00%
22	CAGTAACCCATTCTACATCG	4923	45.00%
23	GGTTTGTCATCAAGCTTAGG	6178	45.00%
24	TGTATGGACTTCAGCAGACA	7151	45.00%
25	TAATTTGGAGGCTCAGGTTC	7516	45.00%
26	TACCGCGATGTTATGCTATA	198	40.00%
27	TATCTAACGCTCTCTTCTGT	594	40.00%
28	TATGTCTAGCTTGATGTTCC	744	40.00%

29	TTTGTCCAGTGGGATCTAAT	2133	40.00%
30	AGTGCACCTGTATGGAATTT	2446	40.00%
31	AGAATCCTCCCTATTAAACC	2606	40.00%
32	TATCTCATCAGCATATCTCC	2870	40.00%
33	GTCTCAGATTCACCATTTGA	2940	40.00%
34	TATCCGGGATCAAGTTGTTT	3229	40.00%
35	CCATTTGTGGTTGAATGGTT	3345	40.00%
36	AGTAAGTGATTCATCTGCCA	4175	40.00%
37	GTACACTTACGTTGTATCCA	4261	40.00%
38	TCTTTATTATCATCTGCGGC	4600	40.00%
39	TCCCAACCATAACCAAATGT	4687	40.00%
40	TAGCTGTTAATGCTCCAACT	4764	40.00%
41	GTATATCCCTACCAACAACA	5007	40.00%
42	TATAGCCCACATCTCTAAGA	5232	40.00%
43	TTTAGGGAAACCCTCATTCA	5486	40.00%
44	TTTGAGCTTCTTTCTTCGTG	6141	40.00%
45	AGCCCAAACTATTACGCATA	6465	40.00%
46	TTTTCATCGCCTGCTATAAC	6682	40.00%
47	TTCAATTCTTCTCTCGTTGC	6730	40.00%
48	CTACCAACTTTACCTAACCA	6832	40.00%

Probes for *Gmm* Negevirus with Quasar 670

Number	Probe (5′→3′)	Probe position	% GC content
1	TAGTGCAAGAAACCTTGGCG	63	50.00%
2	TGTGCACCAAAATCAGTGGG	422	50.00%
3	ACCATATCCAGAAGGAACGG	513	50.00%
4	GTCATACAACGAATGCGCGA	792	50.00%
5	TATCGTAGGGAGAGGAAGCA	1408	50.00%
6	CAGGAGTGGATTGACGGATT	1839	50.00%
7	CGCAATTCGGTACAATGACC	2261	50.00%
8	GTATCAGAAGCGTCAGGATC	2729	50.00%
9	CCTCAACTATAGGAGATGCG	3247	50.00%
10	CTCTGGGAAACTGTATCTGC	3341	50.00%
11	CTCATCACGACTAGAGGGAT	4149	50.00%
12	AACAACCCGGGAATACTCAC	4242	50.00%

13	AACAGATACGCGAGCAGGAT	4305	50.00%
14	CTACCAGAAACAGTAGCGGA	4406	50.00%
15	GAAGCTATGGTACGGTAACC	4490	50.00%
16	TATAAGTCCGAAGCTGCACG	4520	50.00%
17	GGGAATCAGAGCGTGTTGAA	5985	50.00%
18	ACGGGAGATCAGATTCCAAC	6103	50.00%
19	AACACAGTTTGGGAGAGCTT	218	45.00%
20	TGCAAGCGTGAACATTGTCT	586	45.00%
21	TTACAGCATCAGCAGAATCC	841	45.00%
22	GTAGCATGTTCTTGTACACG	1685	45.00%
23	TAAAGTAGCAGCCATGCATC	2004	45.00%
24	AACCCAGAAAGCATTTCAGC	2111	45.00%
25	CAAACACACTCCCATTAGGA	2503	45.00%
26	ACATACTGTATCCAGGAACC	2540	45.00%
27	CTCCTTCACCAGTATAACAG	2596	45.00%
28	TAGTGCAAGAAACCTTGGCG	2693	44.87%
29	TGTGCACCAAAATCAGTGGG	2967	44.62%
30	ACCATATCCAGAAGGAACGG	3223	44.38%
31	GTCATACAACGAATGCGCGA	3272	44.13%
32	TATCGTAGGGAGAGGAAGCA	3298	43.88%
33	CAGGAGTGGATTGACGGATT	3380	43.64%
34	CGCAATTCGGTACAATGACC	3493	43.39%
35	GTATCAGAAGCGTCAGGATC	3554	43.14%
36	CCTCAACTATAGGAGATGCG	4681	42.89%
37	CTCTGGGAAACTGTATCTGC	4988	42.65%
38	CTCATCACGACTAGAGGGAT	5012	42.40%
39	AACAACCCGGGAATACTCAC	5103	42.15%
40	AACAGATACGCGAGCAGGAT	5155	41.90%
41	CTACCAGAAACAGTAGCGGA	5694	41.66%
42	GAAGCTATGGTACGGTAACC	5917	41.41%
43	TATAAGTCCGAAGCTGCACG	6061	41.16%
44	GGGAATCAGAGCGTGTTGAA	6274	40.92%
45	ACGGGAGATCAGATTCCAAC	7295	40.67%
46	AACACAGTTTGGGAGAGCTT	7551	40.42%

47	TGCAAGCGTGAACATTGTCT	7594	40.17%
48	TTACAGCATCAGCAGAATCC	7900	39.93%

Wolbachia probes 488	
**Probe name**	**Probe (5′→3′)**
W1	AATCCGGCCGARCCGACCC
W2	CTTCTGTGAGTACCGTCATTATC
