# Supplementary material for: Characterization and Tissue Tropism of Newly Identified Iflavirus and Negeviruses in Glossina morsitans morsitans Tsetse Flies
Source: Viruses. 2021 Dec 10;13(12):2472. doi: 10.3390/v13122472 (PMC8704047; doi:10.3390/v13122472)
Supplement: Supplementary file 1 [file viruses-13-02472-s001.zip › viruses-1486185-supplementary material-2.pdf]

Characterization and Tissue Tropism of newly identified ifla-virus and negevirus in tsetse flies Glossina morsitans morsitans

Irene K. Meki, Hannah-Isadora Huditz, Anton Strunov, René van Vlugt, Henry M. Kariithi, Mohammadreza Rezapanah, Wolfgang J. Miller, Just M. Vlak, Monique M. van Oers, Adly M.M. Abd-Alla

04/11/2021

set up the working directory and load needed pakages

```
library(ggplot2)
library(lattice)
library(gcookbook)
library(ggfortify)
library(datasets)
library(MASS)
library(survival)
library(rmarkdown)
library(knitr)
library(coxme)

## Loading required package: bdsmatrix

##
## Attaching package: 'bdsmatrix'

## The following object is masked from 'package:base':
##
##     backsolve

library(lme4)

## Loading required package: Matrix

library(nlme)

##
## Attaching package: 'nlme'

## The following object is masked from 'package:lme4':
##
##     lmList

library(tidyverse)

## -- Attaching packages ---------------------------------------
tidyverse 1.3.1 --
```

```
## v tibble  3.1.4     v dplyr   1.0.7
## v tidyr   1.1.3     v stringr 1.4.0
## v readr   2.0.1     v forcats 0.5.1
## v purrr   0.3.4
```

```
## -- Conflicts ------------------------------------------
tidyverse_conflicts() --
## x dplyr::collapse() masks nlme::collapse()
## x tidyr::expand()   masks Matrix::expand()
## x dplyr::filter()   masks stats::filter()
## x dplyr::lag()      masks stats::lag()
## x tidyr::pack()     masks Matrix::pack()
## x dplyr::select()   masks MASS::select()
## x tidyr::unpack()   masks Matrix::unpack()
```

```
library(gapminder)
library(rcompanion)
library(FSA)
```

```
## ## FSA v0.9.1. See citation('FSA') if used in publication.
## ## Run fishR() for related website and fishR('IFAR') for related
book.
```

```
library(stats)
library(RCA)
```

```
## Loading required package: igraph
```

```
##
## Attaching package: 'igraph'
```

```
## The following objects are masked from 'package:dplyr':
##
##     as_data_frame, groups, union
```

```
## The following objects are masked from 'package:purrr':
##
##     compose, simplify
```

```
## The following object is masked from 'package:tidyr':
##
##     crossing
```

```
## The following object is masked from 'package:tibble':
##
##     as_data_frame
```

```
## The following objects are masked from 'package:stats':
##
##     decompose, spectrum
```

```
## The following object is masked from 'package:base':
##
##     union

## Loading required package: gplots

## Registered S3 method overwritten by 'gplots':
##   method         from
##   reorder.factor DescTools

##
## Attaching package: 'gplots'

## The following object is masked from 'package:stats':
##
##     lowess

library(broom)
library(sp)
library(MuMIn)

## Registered S3 methods overwritten by 'MuMIn':
##   method        from
##   formula.coxme coxme
##   logLik.coxme  coxme
##   logLik.lmekin coxme

library(ggpubr)

## Registered S3 methods overwritten by 'car':
##   method       from
##   hist.boot    FSA
##   confint.boot FSA

library(AICcmodavg)

##
## Attaching package: 'AICcmodavg'

## The following objects are masked from 'package:MuMIn':
##
##     AICc, DIC, importance

## The following object is masked from 'package:lme4':
##
##     checkConv

library(car)

## Loading required package: carData

##
## Attaching package: 'car'
```

```
## The following object is masked from 'package:FSA':
##
##     bootCase

## The following object is masked from 'package:dplyr':
##
##     recode

## The following object is masked from 'package:purrr':
##
##     some

library(ggthemes)
```

load the data and prepare it

```
#=====================================
#work with the average of the 3 technical reading
setwd("P:/Hannah/for_Review_meeting")

fig1 <- read.csv("organs_all_avg2.csv")

fig1

##     Target Sample             Tissue Replicate     Virus
Normalized    Sex
## 1      Nege     1A     Salivary_gland         2 Negevirus
77.402705950 Female
## 2      Nege     7A         Fat_bodies         3 Negevirus
39.906292910   Male
## 3      Nege     7A         Fat_bodies         1 Negevirus
39.349594060 Female
## 4      Nege     1A     Salivary_gland         3 Negevirus
29.069334440 Female
## 5      Nege     7A         Fat_bodies         1 Negevirus
20.357023310   Male
## 6      Nege     7A         Fat_bodies         2 Negevirus
19.631228500 Female
## 7      Nege     1A     Salivary_gland         2 Negevirus
18.722890740   Male
## 8      Ifla     7A         Fat_bodies         1 Iflavirus
10.648584190 Female
## 9      Ifla     7A         Fat_bodies         3 Iflavirus
9.314457044 Female
## 10     Nege     3A Malpighian_tubules         1 Negevirus
7.597307635 Female
## 11     Ifla     7A         Fat_bodies         2 Iflavirus
5.013356605 Female
## 12     Nege     1A     Salivary_gland         1 Negevirus
4.376742078   Male
## 13     Ifla     3A Malpighian_tubules         1 Iflavirus
```

```
3.923198149 Female
## 14    Nege     7A          Fat_bodies          2 Negevirus
3.537905209   Male
## 15    Nege     7A          Fat_bodies          3 Negevirus
3.331728226 Female
## 16    Nege     1A      Salivary_gland          3 Negevirus
2.908747434   Male
## 17    Ifla     4A             Mid_gut          3 Iflavirus
2.859857998   Male
## 18    Ifla     8A               Brain          1 Iflavirus
2.764745961 Female
## 19    Nege     8A               Brain          1 Negevirus
2.665310756 Female
## 20    Ifla     1A      Salivary_gland          1 Iflavirus
1.918755308 Female
## 21    Ifla     4A             Mid_gut          3 Iflavirus
1.825265012 Female
## 22    Ifla     6A              Testes          3 Iflavirus
1.794807611   Male
## 23    Ifla     8A               Brain          2 Iflavirus
1.786912992 Female
## 24    Nege     8A               Brain          2 Negevirus
1.773831321 Female
## 25    Ifla     6A              Testes          1 Iflavirus
1.697621456   Male
## 26    Nege     9A         Milk_glands          2 Negevirus
1.412966980 Female
## 27    Ifla     3A Malpighian_tubules          3 Iflavirus
1.411609258 Female
## 28    Ifla     9A         Milk_glands          2 Iflavirus
1.385602181 Female
## 29    Ifla     6A              Testes          2 Iflavirus
1.320664580   Male
## 30    Ifla     3A Malpighian_tubules          2 Iflavirus
1.301843553   Male
## 31    Ifla     7A          Fat_bodies          3 Iflavirus
1.269430000   Male
## 32    Ifla     4A             Mid_gut          1 Iflavirus
1.211337107 Female
## 33    Ifla     1A      Salivary_gland          3 Iflavirus
1.209748099   Male
## 34    Ifla     8A               Brain          3 Iflavirus
1.207589381   Male
## 35    Ifla     9A         Milk_glands          3 Iflavirus
1.178765779 Female
## 36    Nege     5A           Front_gut          1 Negevirus
1.141186814 Female
## 37    Nege     8A               Brain          1 Negevirus
1.060610153   Male
## 38    Nege     6A              Testes          3 Negevirus
```

```
1.048261614   Male
## 39    Ifla     8A              Brain          3 Iflavirus
1.023812750 Female
## 40    Ifla     9A        Milk_glands          1 Iflavirus
1.021891196 Female
## 41    Ifla     8A              Brain          1 Iflavirus
1.021211123   Male
## 42    Ifla     4A            Mid_gut          2 Iflavirus
1.006193603   Male
## 43    Nege     6A              Ovary          1 Negevirus
0.996945359 Female
## 44    Nege     6A            Testes          1 Negevirus
0.981968623   Male
## 45    Ifla     1A     Salivary_gland          3 Iflavirus
0.967271882 Female
## 46    Nege     4A            Mid_gut          3 Negevirus
0.944433541   Male
## 47    Ifla     7A         Fat_bodies          2 Iflavirus
0.930867859   Male
## 48    Ifla     8A              Brain          2 Iflavirus
0.864353004   Male
## 49    Nege     5A          Front_gut          3 Negevirus
0.852571665   Male
## 50    Ifla     1A     Salivary_gland          2 Iflavirus
0.817396209 Female
## 51    Ifla     5A          Front_gut          3 Iflavirus
0.794423167   Male
## 52    Ifla     3A Malpighian_tubules          3 Iflavirus
0.754561236   Male
## 53    Nege     4A            Mid_gut          1 Negevirus
0.751201684 Female
## 54    Ifla     4A            Mid_gut          2 Iflavirus
0.719587999 Female
## 55    Ifla     1A     Salivary_gland          2 Iflavirus
0.659558977   Male
## 56    Nege     2A           Hind_gut          1 Negevirus
0.596460970 Female
## 57    Nege     9A        Milk_glands          1 Negevirus
0.579908913 Female
## 58    Ifla     5A          Front_gut          2 Iflavirus
0.489249866   Male
## 59    Ifla     5A          Front_gut          3 Iflavirus
0.474846292 Female
## 60    Nege     8A              Brain          3 Negevirus
0.454187308   Male
## 61    Ifla     5A          Front_gut          1 Iflavirus
0.428891911 Female
## 62    Nege     4A            Mid_gut          1 Negevirus
0.417650378   Male
## 63    Ifla     7A         Fat_bodies          1 Iflavirus
```

```
0.402634738   Male
## 64    Ifla     3A Malpighian_tubules         1 Iflavirus
0.371600886   Male
## 65    Nege     9A        Milk_glands         3 Negevirus
0.371587771 Female
## 66    Ifla     4A            Mid_gut         1 Iflavirus
0.365671927   Male
## 67    Nege     1A     Salivary_gland         1 Negevirus
0.358316060 Female
## 68    Ifla     2A           Hind_gut         1 Iflavirus
0.355361037 Female
## 69    Ifla     3A Malpighian_tubules         2 Iflavirus
0.354209947 Female
## 70    Nege     6A             Testes         2 Negevirus
0.352838330   Male
## 71    Nege     6A              Ovary         2 Negevirus
0.352644646 Female
## 72    Nege     4A            Mid_gut         2 Negevirus
0.321005816 Female
## 73    Ifla     2A           Hind_gut         1 Iflavirus
0.272778994   Male
## 74    Nege     2A           Hind_gut         1 Negevirus
0.246349379   Male
## 75    Nege     3A Malpighian_tubules         3 Negevirus
0.225166941   Male
## 76    Nege     5A          Front_gut         1 Negevirus
0.196212798   Male
## 77    Nege     8A              Brain         2 Negevirus
0.179375639   Male
## 78    Ifla     2A           Hind_gut         3 Iflavirus
0.170321852   Male
## 79    Nege     3A Malpighian_tubules         2 Negevirus
0.159920548 Female
## 80    Ifla     2A           Hind_gut         2 Iflavirus
0.146907611 Female
## 81    Ifla     2A           Hind_gut         3 Iflavirus
0.130858443 Female
## 82    Ifla     1A     Salivary_gland         1 Iflavirus
0.117672441   Male
## 83    Ifla     2A           Hind_gut         2 Iflavirus
0.109334722   Male
## 84    Ifla     5A          Front_gut         1 Iflavirus
0.107608329   Male
## 85    Nege     3A Malpighian_tubules         1 Negevirus
0.103543820   Male
## 86    Nege     5A          Front_gut         2 Negevirus
0.100733703 Female
## 87    Ifla     5A          Front_gut         2 Iflavirus
0.099571356 Female
## 88    Nege     4A            Mid_gut         3 Negevirus
```

```
0.094092464 Female
## 89    Nege     2A           Hind_gut         2 Negevirus
0.089570912 Female
## 90    Nege     5A          Front_gut         2 Negevirus
0.071801559   Male
## 91    Nege     8A              Brain         3 Negevirus
0.057932219 Female
## 92    Nege     3A Malpighian_tubules         3 Negevirus
0.042103969 Female
## 93    Nege     5A          Front_gut         3 Negevirus
0.039952549 Female
## 94    Nege     6A              Ovary         3 Negevirus
0.032297897 Female
## 95    Nege     4A            Mid_gut         2 Negevirus
0.028138818   Male
## 96    Nege     2A           Hind_gut         3 Negevirus
0.023073452   Male
## 97    Ifla     6A              Ovary         3 Iflavirus
0.021062121 Female
## 98    Ifla     6A              Ovary         1 Iflavirus
0.017170381 Female
## 99    Nege     3A Malpighian_tubules         2 Negevirus
0.015372781   Male
## 100   Nege     2A           Hind_gut         3 Negevirus
0.012167280 Female
## 101   Ifla     6A              Ovary         2 Iflavirus
0.007298148 Female
## 102   Nege     2A           Hind_gut         2 Negevirus
0.003874746   Male

str(fig1)

## 'data.frame':    102 obs. of  7 variables:
##  $ Target    : chr  "Nege" "Nege" "Nege" "Nege" ...
##  $ Sample    : chr  "1A" "7A" "7A" "1A" ...
##  $ Tissue    : chr  "Salivary_gland" "Fat_bodies" "Fat_bodies"
"Salivary_gland" ...
##  $ Replicate : int  2 3 1 3 1 2 2 1 3 1 ...
##  $ Virus     : chr  "Negevirus" "Negevirus" "Negevirus" "Negevirus"
...
##  $ Normalized: num  77.4 39.9 39.3 29.1 20.4 ...
##  $ Sex       : chr  "Female" "Male" "Female" "Female" ...

#qpcr1$Normalized_log_SQ<-as.numeric(qpcr1$Normalized_log_SQ)
attach(fig1)
head(fig1)

##   Target Sample         Tissue Replicate     Virus Normalized    Sex
## 1   Nege     1A Salivary_gland         2 Negevirus   77.40271 Female
## 2   Nege     7A     Fat_bodies         3 Negevirus   39.90629   Male
## 3   Nege     7A     Fat_bodies         1 Negevirus   39.34959 Female
```

```
## 4    Nege      1A  Salivary_gland         3 Negevirus   29.06933 Female
## 5    Nege      7A      Fat_bodies         1 Negevirus   20.35702   Male
## 6    Nege      7A      Fat_bodies         2 Negevirus   19.63123 Female

qpcr=na.omit(fig1)
```

Prepare fig 1 A and B

```
#produce figure
summary(fig1)

##     Target              Sample              Tissue
Replicate
##  Length:102         Length:102         Length:102         Min.   :1
##  Class :character   Class :character   Class :character   1st Qu.:1
##  Mode  :character   Mode  :character   Mode  :character   Median :2
##                                                           Mean   :2
##                                                           3rd Qu.:3
##                                                           Max.   :3
##     Virus             Normalized            Sex
##  Length:102         Min.   : 0.00387   Length:102
##  Class :character   1st Qu.: 0.18358   Class :character
##  Mode  :character   Median : 0.77449   Mode  :character
##                     Mean   : 3.48446
##                     3rd Qu.: 1.41263
##                     Max.   :77.40271

#to produce figure 6A
figs6avg<-ggplot(fig1,aes(x=Tissue,y=Normalized, fill=Tissue)) +
  geom_boxplot() + geom_jitter(width=0.1,alpha=0.2) +
  facet_grid(Sex~Virus) + ylim(0,80)
figs6avg
```

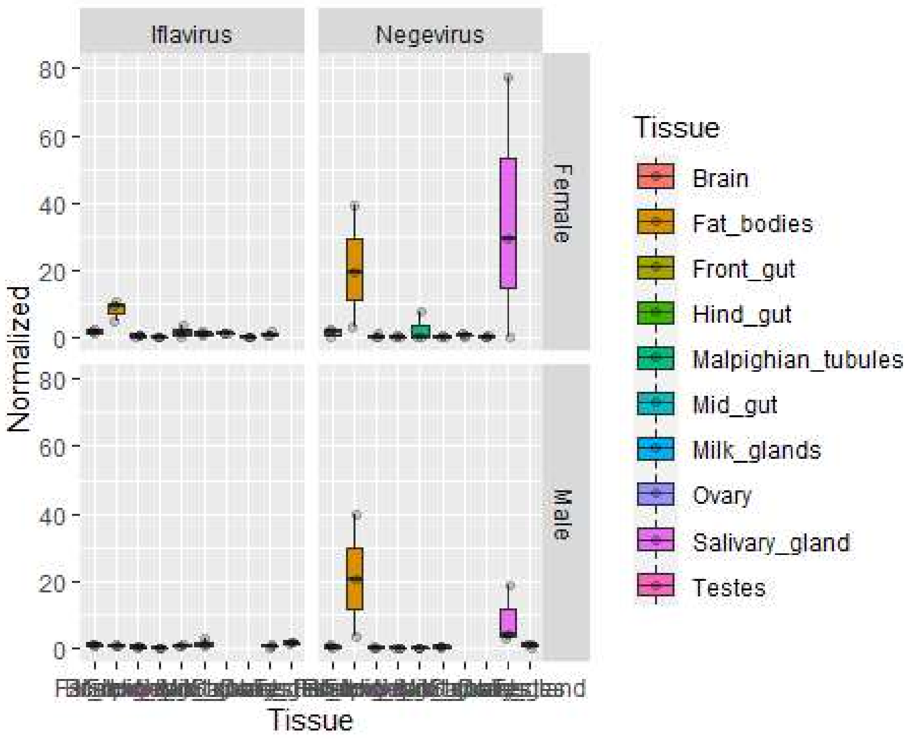


```
tiff("figs6avg1.tiff", width = 6, height = 6, units = 'in', res = 300)
plot(figs6avg+theme_tufte() + theme(axis.line = element_line(size = 1,
colour = "black")) +
theme(axis.text.x=element_blank(),axis.ticks.x=element_blank()) +
theme(legend.title = element_text(face = "bold")) + theme(legend.text =
element_text(face = "italic"))) + xlab(expression(bold("Tissues"))) +
ylab(expression (paste (bold("Normalized  "), bold("density"))))
dev.off()

## png
##   2

#To produce the supllementary figure 6B
figs6avg<-ggplot(fig1,aes(x=Tissue,y=Normalized, fill=Tissue)) +
  geom_boxplot() + geom_jitter(width=0.1,alpha=0.2) +
  facet_grid(Sex~Virus) + ylim(0,5)
figs6avg

## Warning: Removed 11 rows containing non-finite values
(stat_boxplot).

## Warning: Removed 11 rows containing missing values (geom_point).
```

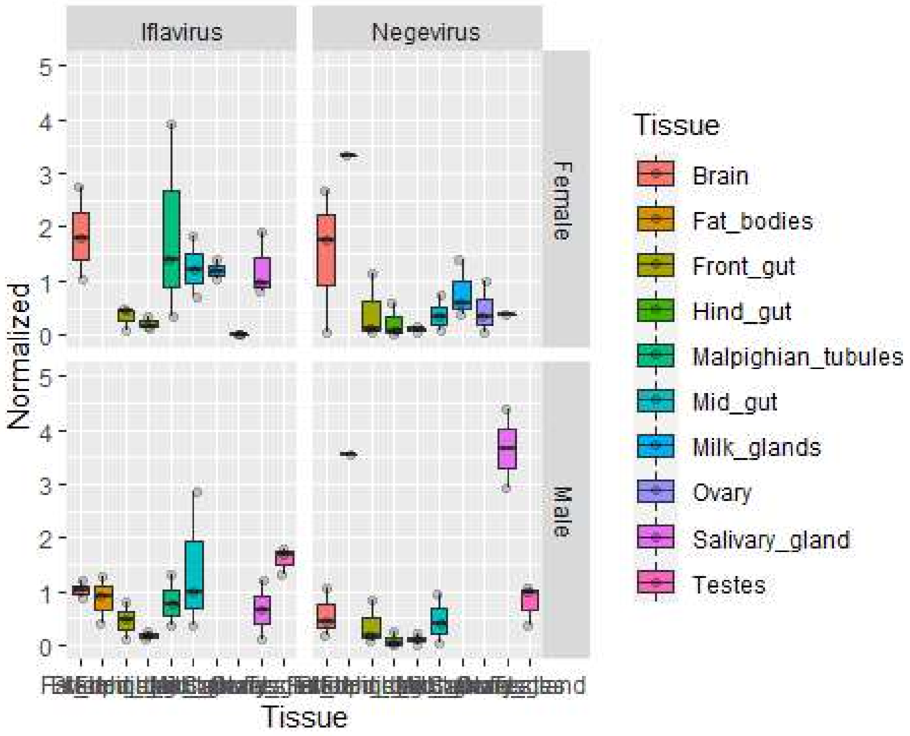


```
tiff("figs6avg1.tiff", width = 6, height = 6, units = 'in', res = 300)
plot(figs6avg+theme_tufte() + theme(axis.line = element_line(size = 1,
colour = "black")) +
theme(axis.text.x=element_blank(),axis.ticks.x=element_blank()) +
theme(legend.title = element_text(face = "bold")) + theme(legend.text =
element_text(face = "italic"))) + xlab(expression(bold("Tissues"))) +
ylab(expression (paste (bold("Normalized  "), bold("density")))))

## Warning: Removed 11 rows containing non-finite values
(stat_boxplot).

## Warning: Removed 11 rows containing missing values (geom_point).

## Warning: Removed 11 rows containing non-finite values
(stat_boxplot).

## Warning: Removed 11 rows containing missing values (geom_point).

dev.off()

## png
##   2

#difference between tiussue regardsles the virus or the sex
model1<-glm(Normalized ~ Tissue, data = fig1)
summary(model1)
```

```
##
## Call:
## glm(formula = Normalized ~ Tissue, data = fig1)
##
## Deviance Residuals:
##      Min        1Q    Median        3Q       Max
## -12.405    -0.950    -0.177     0.184    65.859
##
## Coefficients:
##                             Estimate Std. Error t value Pr(>|t|)
## (Intercept)                  1.23832    2.67881   0.462  0.64498
## TissueFat_bodies            11.56944    3.78841   3.054  0.00295 **
## TissueFront_gut             -0.83857    3.78841  -0.221  0.82531
## TissueHind_gut              -1.05857    3.78841  -0.279  0.78055
## TissueMalpighian_tubules     0.11671    3.78841   0.031  0.97549
## TissueMid_gut               -0.35962    3.78841  -0.095  0.92458
## TissueMilk_glands           -0.24654    4.63983  -0.053  0.95774
## TissueOvary                 -1.00042    4.63983  -0.216  0.82976
## TissueSalivary_gland        10.30577    3.78841   2.720  0.00780 **
## TissueTestes                -0.03896    4.63983  -0.008  0.99332
## ---
## Signif. codes:  0 '***' 0.001 '**' 0.01 '*' 0.05 '.' 0.1 ' ' 1
##
## (Dispersion parameter for gaussian family taken to be 86.11222)
##
##     Null deviance: 10318.4  on 101  degrees of freedom
## Residual deviance:  7922.3  on  92  degrees of freedom
## AIC: 755.42
##
## Number of Fisher Scoring iterations: 2
```

```
Anova(model1)
```

```
## Analysis of Deviance Table (Type II tests)
##
## Response: Normalized
##        LR Chisq Df Pr(>Chisq)
## Tissue   27.825  9    0.00102 **
## ---
## Signif. codes:  0 '***' 0.001 '**' 0.01 '*' 0.05 '.' 0.1 ' ' 1
```

statistical analysis for Iflavirus

```
#work with iflavirus alone

fig1ifla <- subset(fig1, Virus=="Iflavirus")
fig1ifla
```

```
##     Target Sample              Tissue Replicate     Virus
Normalized     Sex
## 8      Ifla      7A          Fat_bodies         1 Iflavirus
```

```
10.648584190 Female
## 9     Ifla     7A          Fat_bodies         3 Iflavirus
9.314457044 Female
## 11    Ifla     7A          Fat_bodies         2 Iflavirus
5.013356605 Female
## 13    Ifla     3A Malpighian_tubules         1 Iflavirus
3.923198149 Female
## 17    Ifla     4A             Mid_gut         3 Iflavirus
2.859857998   Male
## 18    Ifla     8A               Brain         1 Iflavirus
2.764745961 Female
## 20    Ifla     1A     Salivary_gland         1 Iflavirus
1.918755308 Female
## 21    Ifla     4A             Mid_gut         3 Iflavirus
1.825265012 Female
## 22    Ifla     6A              Testes         3 Iflavirus
1.794807611   Male
## 23    Ifla     8A               Brain         2 Iflavirus
1.786912992 Female
## 25    Ifla     6A              Testes         1 Iflavirus
1.697621456   Male
## 27    Ifla     3A Malpighian_tubules         3 Iflavirus
1.411609258 Female
## 28    Ifla     9A         Milk_glands         2 Iflavirus
1.385602181 Female
## 29    Ifla     6A              Testes         2 Iflavirus
1.320664580   Male
## 30    Ifla     3A Malpighian_tubules         2 Iflavirus
1.301843553   Male
## 31    Ifla     7A          Fat_bodies         3 Iflavirus
1.269430000   Male
## 32    Ifla     4A             Mid_gut         1 Iflavirus
1.211337107 Female
## 33    Ifla     1A     Salivary_gland         3 Iflavirus
1.209748099   Male
## 34    Ifla     8A               Brain         3 Iflavirus
1.207589381   Male
## 35    Ifla     9A         Milk_glands         3 Iflavirus
1.178765779 Female
## 39    Ifla     8A               Brain         3 Iflavirus
1.023812750 Female
## 40    Ifla     9A         Milk_glands         1 Iflavirus
1.021891196 Female
## 41    Ifla     8A               Brain         1 Iflavirus
1.021211123   Male
## 42    Ifla     4A             Mid_gut         2 Iflavirus
1.006193603   Male
## 45    Ifla     1A     Salivary_gland         3 Iflavirus
0.967271882 Female
## 47    Ifla     7A          Fat_bodies         2 Iflavirus
```

```
0.930867859   Male
## 48    Ifla     8A              Brain          2 Iflavirus
0.864353004   Male
## 50    Ifla     1A     Salivary_gland          2 Iflavirus
0.817396209 Female
## 51    Ifla     5A          Front_gut          3 Iflavirus
0.794423167   Male
## 52    Ifla     3A Malpighian_tubules          3 Iflavirus
0.754561236   Male
## 54    Ifla     4A            Mid_gut          2 Iflavirus
0.719587999 Female
## 55    Ifla     1A     Salivary_gland          2 Iflavirus
0.659558977   Male
## 58    Ifla     5A          Front_gut          2 Iflavirus
0.489249866   Male
## 59    Ifla     5A          Front_gut          3 Iflavirus
0.474846292 Female
## 61    Ifla     5A          Front_gut          1 Iflavirus
0.428891911 Female
## 63    Ifla     7A         Fat_bodies          1 Iflavirus
0.402634738   Male
## 64    Ifla     3A Malpighian_tubules          1 Iflavirus
0.371600886   Male
## 66    Ifla     4A            Mid_gut          1 Iflavirus
0.365671927   Male
## 68    Ifla     2A           Hind_gut          1 Iflavirus
0.355361037 Female
## 69    Ifla     3A Malpighian_tubules          2 Iflavirus
0.354209947 Female
## 73    Ifla     2A           Hind_gut          1 Iflavirus
0.272778994   Male
## 78    Ifla     2A           Hind_gut          3 Iflavirus
0.170321852   Male
## 80    Ifla     2A           Hind_gut          2 Iflavirus
0.146907611 Female
## 81    Ifla     2A           Hind_gut          3 Iflavirus
0.130858443 Female
## 82    Ifla     1A     Salivary_gland          1 Iflavirus
0.117672441   Male
## 83    Ifla     2A           Hind_gut          2 Iflavirus
0.109334722   Male
## 84    Ifla     5A          Front_gut          1 Iflavirus
0.107608329   Male
## 87    Ifla     5A          Front_gut          2 Iflavirus
0.099571356 Female
## 97    Ifla     6A              Ovary          3 Iflavirus
0.021062121 Female
## 98    Ifla     6A              Ovary          1 Iflavirus
0.017170381 Female
```

```
## 101   Ifla     6A              Ovary         2 Iflavirus
0.007298148 Female

#difference between tissue regardsless the sex for iflavirus
model1<-glm(Normalized ~ Tissue, data = fig1ifla)
summary(model1)

##
## Call:
## glm(formula = Normalized ~ Tissue, data = fig1ifla)
##
## Deviance Residuals:
##     Min       1Q   Median       3Q      Max
## -4.1939  -0.3123  -0.0273   0.1740   6.0520
##
## Coefficients:
##                            Estimate Std. Error t value Pr(>|t|)
## (Intercept)                 1.44477    0.69577   2.077  0.04416 *
## TissueFat_bodies            3.15178    0.98397   3.203  0.00263 **
## TissueFront_gut            -1.04567    0.98397  -1.063  0.29413
## TissueHind_gut             -1.24718    0.98397  -1.267  0.21213
## TissueMalpighian_tubules -0.09193    0.98397  -0.093  0.92602
## TissueMid_gut              -0.11345    0.98397  -0.115  0.90877
## TissueMilk_glands          -0.24935    1.20511  -0.207  0.83710
## TissueOvary                -1.42959    1.20511  -1.186  0.24234
## TissueSalivary_gland       -0.49637    0.98397  -0.504  0.61664
## TissueTestes                0.15959    1.20511   0.132  0.89529
## ---
## Signif. codes:  0 '***' 0.001 '**' 0.01 '*' 0.05 '.' 0.1 ' ' 1
##
## (Dispersion parameter for gaussian family taken to be 2.904571)
##
##     Null deviance: 202.33  on 50  degrees of freedom
## Residual deviance: 119.09  on 41  degrees of freedom
## AIC: 209.98
##
## Number of Fisher Scoring iterations: 2

Anova(model1)

## Analysis of Deviance Table (Type II tests)
##
## Response: Normalized
##        LR Chisq Df Pr(>Chisq)
## Tissue   28.658  9  0.0007399 ***
## ---
## Signif. codes:  0 '***' 0.001 '**' 0.01 '*' 0.05 '.' 0.1 ' ' 1

#difference between sex regardsless the tissues for iflavirus
model1<-glm(Normalized ~ Sex, data = fig1ifla)
summary(model1)
```

```
##
## Call:
## glm(formula = Normalized ~ Sex, data = fig1ifla)
##
## Deviance Residuals:
##     Min       1Q   Median       3Q      Max
## -1.8064  -0.8191  -0.4281   0.2353   8.8349
##
## Coefficients:
##             Estimate Std. Error t value Pr(>|t|)
## (Intercept)   1.8137     0.3802   4.770 1.69e-05 ***
## SexMale      -0.9345     0.5542  -1.686   0.0981 .
## ---
## Signif. codes:  0 '***' 0.001 '**' 0.01 '*' 0.05 '.' 0.1 ' ' 1
##
## (Dispersion parameter for gaussian family taken to be 3.902679)
##
##     Null deviance: 202.33  on 50  degrees of freedom
## Residual deviance: 191.23  on 49  degrees of freedom
## AIC: 218.14
##
## Number of Fisher Scoring iterations: 2
```

```
Anova(model1)
```

```
## Analysis of Deviance Table (Type II tests)
##
## Response: Normalized
##     LR Chisq Df Pr(>Chisq)
## Sex   2.8432  1    0.09176 .
## ---
## Signif. codes:  0 '***' 0.001 '**' 0.01 '*' 0.05 '.' 0.1 ' ' 1
```

```
#==============================================
#for male
fig1iflam <- subset(fig1ifla, Sex=="Male")
fig1iflam
```

```
##    Target Sample             Tissue Replicate     Virus Normalized
Sex
## 17   Ifla     4A            Mid_gut         3 Iflavirus  2.8598580
Male
## 22   Ifla     6A             Testes         3 Iflavirus  1.7948076
Male
## 25   Ifla     6A             Testes         1 Iflavirus  1.6976215
Male
## 29   Ifla     6A             Testes         2 Iflavirus  1.3206646
Male
## 30   Ifla     3A Malpighian_tubules         2 Iflavirus  1.3018436
Male
## 31   Ifla     7A         Fat_bodies         3 Iflavirus  1.2694300
```

```
Male
## 33    Ifla      1A      Salivary_gland         3 Iflavirus  1.2097481
Male
## 34    Ifla      8A               Brain         3 Iflavirus  1.2075894
Male
## 41    Ifla      8A               Brain         1 Iflavirus  1.0212111
Male
## 42    Ifla      4A             Mid_gut         2 Iflavirus  1.0061936
Male
## 47    Ifla      7A          Fat_bodies         2 Iflavirus  0.9308679
Male
## 48    Ifla      8A               Brain         2 Iflavirus  0.8643530
Male
## 51    Ifla      5A           Front_gut         3 Iflavirus  0.7944232
Male
## 52    Ifla      3A Malpighian_tubules         3 Iflavirus  0.7545612
Male
## 55    Ifla      1A      Salivary_gland         2 Iflavirus  0.6595590
Male
## 58    Ifla      5A           Front_gut         2 Iflavirus  0.4892499
Male
## 63    Ifla      7A          Fat_bodies         1 Iflavirus  0.4026347
Male
## 64    Ifla      3A Malpighian_tubules         1 Iflavirus  0.3716009
Male
## 66    Ifla      4A             Mid_gut         1 Iflavirus  0.3656719
Male
## 73    Ifla      2A            Hind_gut         1 Iflavirus  0.2727790
Male
## 78    Ifla      2A            Hind_gut         3 Iflavirus  0.1703219
Male
## 82    Ifla      1A      Salivary_gland         1 Iflavirus  0.1176724
Male
## 83    Ifla      2A            Hind_gut         2 Iflavirus  0.1093347
Male
## 84    Ifla      5A           Front_gut         1 Iflavirus  0.1076083
Male

fig1iflam$Tissue <- as.factor(fig1iflam$Tissue)
fig1iflam$Tissue <- relevel(fig1iflam$Tissue, ref= "Brain")
model1<-glm(Normalized ~ Tissue, data = fig1iflam)
summary(model1)

##
## Call:
## glm(formula = Normalized ~ Tissue, data = fig1iflam)
##
## Deviance Residuals:
##      Min       1Q   Median       3Q      Max
## -1.0449  -0.3018  -0.0063   0.1800   1.4493
```

```
##
## Coefficients:
##                            Estimate Std. Error t value Pr(>|t|)
## (Intercept)                  1.0311     0.3293   3.131  0.00645 **
## TissueFat_bodies            -0.1634     0.4657  -0.351  0.73027
## TissueFront_gut             -0.5673     0.4657  -1.218  0.24087
## TissueHind_gut              -0.8469     0.4657  -1.818  0.08777 .
## TissueMalpighian_tubules  -0.2217     0.4657  -0.476  0.64047
## TissueMid_gut                0.3795     0.4657   0.815  0.42710
## TissueSalivary_gland        -0.3687     0.4657  -0.792  0.44012
## TissueTestes                 0.5733     0.4657   1.231  0.23612
## ---
## Signif. codes:  0 '***' 0.001 '**' 0.01 '*' 0.05 '.' 0.1 ' ' 1
##
## (Dispersion parameter for gaussian family taken to be 0.3253694)
##
##     Null deviance: 9.8230  on 23  degrees of freedom
## Residual deviance: 5.2059  on 16  degrees of freedom
## AIC: 49.431
##
## Number of Fisher Scoring iterations: 2
```

```
Anova(model1)
```

```
## Analysis of Deviance Table (Type II tests)
##
## Response: Normalized
##        LR Chisq Df Pr(>Chisq)
## Tissue    14.19  7     0.0479 *
## ---
## Signif. codes:  0 '***' 0.001 '**' 0.01 '*' 0.05 '.' 0.1 ' ' 1
```

```
fig1iflam$Tissue <- relevel(fig1iflam$Tissue, ref= "Mid_gut")
model1<-glm(Normalized ~ Tissue, data = fig1iflam)
summary(model1)
```

```
##
## Call:
## glm(formula = Normalized ~ Tissue, data = fig1iflam)
##
## Deviance Residuals:
##     Min       1Q   Median       3Q      Max
## -1.0449  -0.3018  -0.0063   0.1800   1.4493
##
## Coefficients:
##                            Estimate Std. Error t value Pr(>|t|)
## (Intercept)                  1.4106     0.3293   4.283  0.00057 ***
## TissueBrain                 -0.3795     0.4657  -0.815  0.42710
## TissueFat_bodies            -0.5429     0.4657  -1.166  0.26080
## TissueFront_gut             -0.9468     0.4657  -2.033  0.05900 .
## TissueHind_gut              -1.2264     0.4657  -2.633  0.01807 *
```

```
## TissueMalpighian_tubules  -0.6012     0.4657  -1.291  0.21507
## TissueSalivary_gland      -0.7482     0.4657  -1.607  0.12770
## TissueTestes               0.1938     0.4657   0.416  0.68287
## ---
## Signif. codes:  0 '***' 0.001 '**' 0.01 '*' 0.05 '.' 0.1 ' ' 1
##
## (Dispersion parameter for gaussian family taken to be 0.3253694)
##
##     Null deviance: 9.8230  on 23  degrees of freedom
## Residual deviance: 5.2059  on 16  degrees of freedom
## AIC: 49.431
##
## Number of Fisher Scoring iterations: 2
```

```
Anova(model1)
```

```
## Analysis of Deviance Table (Type II tests)
##
## Response: Normalized
##        LR Chisq Df Pr(>Chisq)
## Tissue    14.19  7     0.0479 *
## ---
## Signif. codes:  0 '***' 0.001 '**' 0.01 '*' 0.05 '.' 0.1 ' ' 1
```

```
fig1iflam$Tissue <- relevel(fig1iflam$Tissue, ref= "Testes")
model1<-glm(Normalized ~ Tissue, data = fig1iflam)
summary(model1)
```

```
##
## Call:
## glm(formula = Normalized ~ Tissue, data = fig1iflam)
##
## Deviance Residuals:
##     Min       1Q   Median       3Q      Max
## -1.0449  -0.3018  -0.0063   0.1800   1.4493
##
## Coefficients:
##                           Estimate Std. Error t value Pr(>|t|)
## (Intercept)                 1.6044     0.3293   4.872  0.00017 ***
## TissueMid_gut              -0.1938     0.4657  -0.416  0.68287
## TissueBrain                -0.5733     0.4657  -1.231  0.23612
## TissueFat_bodies           -0.7367     0.4657  -1.582  0.13325
## TissueFront_gut            -1.1406     0.4657  -2.449  0.02622 *
## TissueHind_gut             -1.4202     0.4657  -3.049  0.00765 **
## TissueMalpighian_tubules   -0.7950     0.4657  -1.707  0.10715
## TissueSalivary_gland       -0.9420     0.4657  -2.023  0.06015 .
## ---
## Signif. codes:  0 '***' 0.001 '**' 0.01 '*' 0.05 '.' 0.1 ' ' 1
##
## (Dispersion parameter for gaussian family taken to be 0.3253694)
##
```

```
##     Null deviance: 9.8230  on 23  degrees of freedom
## Residual deviance: 5.2059  on 16  degrees of freedom
## AIC: 49.431
##
## Number of Fisher Scoring iterations: 2

Anova(model1)

## Analysis of Deviance Table (Type II tests)
##
## Response: Normalized
##        LR Chisq Df Pr(>Chisq)
## Tissue    14.19  7     0.0479 *
## ---
## Signif. codes:  0 '***' 0.001 '**' 0.01 '*' 0.05 '.' 0.1 ' ' 1

fig1iflam$Tissue <- relevel(fig1iflam$Tissue, ref= "Fat_bodies")
model1<-glm(Normalized ~ Tissue, data = fig1iflam)
summary(model1)

##
## Call:
## glm(formula = Normalized ~ Tissue, data = fig1iflam)
##
## Deviance Residuals:
##     Min       1Q   Median       3Q      Max
## -1.0449  -0.3018  -0.0063   0.1800   1.4493
##
## Coefficients:
##                            Estimate Std. Error t value Pr(>|t|)
## (Intercept)                 0.86764    0.32933   2.635    0.018 *
## TissueTestes                0.73672    0.46574   1.582    0.133
## TissueMid_gut               0.54293    0.46574   1.166    0.261
## TissueBrain                 0.16341    0.46574   0.351    0.730
## TissueFront_gut            -0.40388    0.46574  -0.867    0.399
## TissueHind_gut             -0.68350    0.46574  -1.468    0.162
## TissueMalpighian_tubules -0.05831    0.46574  -0.125    0.902
## TissueSalivary_gland       -0.20532    0.46574  -0.441    0.665
## ---
## Signif. codes:  0 '***' 0.001 '**' 0.01 '*' 0.05 '.' 0.1 ' ' 1
##
## (Dispersion parameter for gaussian family taken to be 0.3253694)
##
##     Null deviance: 9.8230  on 23  degrees of freedom
## Residual deviance: 5.2059  on 16  degrees of freedom
## AIC: 49.431
##
## Number of Fisher Scoring iterations: 2

Anova(model1)
```

```
## Analysis of Deviance Table (Type II tests)
##
## Response: Normalized
##        LR Chisq Df Pr(>Chisq)
## Tissue    14.19  7     0.0479 *
## ---
## Signif. codes:  0 '***' 0.001 '**' 0.01 '*' 0.05 '.' 0.1 ' ' 1
```

```
fig1iflam$Tissue <- relevel(fig1iflam$Tissue, ref= "Salivary_gland")
model1<-glm(Normalized ~ Tissue, data = fig1iflam)
summary(model1)
```

```
##
## Call:
## glm(formula = Normalized ~ Tissue, data = fig1iflam)
##
## Deviance Residuals:
##     Min       1Q   Median       3Q      Max
## -1.0449  -0.3018  -0.0063   0.1800   1.4493
##
## Coefficients:
##                            Estimate Std. Error t value Pr(>|t|)
## (Intercept)                  0.6623     0.3293   2.011   0.0615 .
## TissueFat_bodies             0.2053     0.4657   0.441   0.6652
## TissueTestes                 0.9420     0.4657   2.023   0.0602 .
## TissueMid_gut                0.7482     0.4657   1.607   0.1277
## TissueBrain                  0.3687     0.4657   0.792   0.4401
## TissueFront_gut             -0.1986     0.4657  -0.426   0.6755
## TissueHind_gut              -0.4782     0.4657  -1.027   0.3198
## TissueMalpighian_tubules     0.1470     0.4657   0.316   0.7564
## ---
## Signif. codes:  0 '***' 0.001 '**' 0.01 '*' 0.05 '.' 0.1 ' ' 1
##
## (Dispersion parameter for gaussian family taken to be 0.3253694)
##
##     Null deviance: 9.8230  on 23  degrees of freedom
## Residual deviance: 5.2059  on 16  degrees of freedom
## AIC: 49.431
##
## Number of Fisher Scoring iterations: 2
```

```
Anova(model1)
```

```
## Analysis of Deviance Table (Type II tests)
##
## Response: Normalized
##        LR Chisq Df Pr(>Chisq)
## Tissue    14.19  7     0.0479 *
## ---
## Signif. codes:  0 '***' 0.001 '**' 0.01 '*' 0.05 '.' 0.1 ' ' 1
```

```
fig1iflam$Tissue <- relevel(fig1iflam$Tissue, ref= "Front_gut")
model1<-glm(Normalized ~ Tissue, data = fig1iflam)
summary(model1)

##
## Call:
## glm(formula = Normalized ~ Tissue, data = fig1iflam)
##
## Deviance Residuals:
##     Min       1Q   Median       3Q      Max
## -1.0449  -0.3018  -0.0063   0.1800   1.4493
##
## Coefficients:
##                             Estimate Std. Error t value Pr(>|t|)
## (Intercept)                   0.4638     0.3293   1.408   0.1782
## TissueSalivary_gland          0.1986     0.4657   0.426   0.6755
## TissueFat_bodies              0.4039     0.4657   0.867   0.3987
## TissueTestes                  1.1406     0.4657   2.449   0.0262 *
## TissueMid_gut                 0.9468     0.4657   2.033   0.0590 .
## TissueBrain                   0.5673     0.4657   1.218   0.2409
## TissueHind_gut               -0.2796     0.4657  -0.600   0.5567
## TissueMalpighian_tubules      0.3456     0.4657   0.742   0.4688
## ---
## Signif. codes:  0 '***' 0.001 '**' 0.01 '*' 0.05 '.' 0.1 ' ' 1
##
## (Dispersion parameter for gaussian family taken to be 0.3253694)
##
##     Null deviance: 9.8230  on 23  degrees of freedom
## Residual deviance: 5.2059  on 16  degrees of freedom
## AIC: 49.431
##
## Number of Fisher Scoring iterations: 2

Anova(model1)

## Analysis of Deviance Table (Type II tests)
##
## Response: Normalized
##        LR Chisq Df Pr(>Chisq)
## Tissue    14.19  7     0.0479 *
## ---
## Signif. codes:  0 '***' 0.001 '**' 0.01 '*' 0.05 '.' 0.1 ' ' 1

fig1iflam$Tissue <- relevel(fig1iflam$Tissue, ref= "Hind_gut")
model1<-glm(Normalized ~ Tissue, data = fig1iflam)
summary(model1)

##
## Call:
## glm(formula = Normalized ~ Tissue, data = fig1iflam)
##
```

```
## Deviance Residuals:
##     Min       1Q   Median       3Q      Max
## -1.0449  -0.3018  -0.0063   0.1800   1.4493
##
## Coefficients:
##                          Estimate Std. Error t value Pr(>|t|)
## (Intercept)                0.1841     0.3293   0.559  0.58380
## TissueFront_gut            0.2796     0.4657   0.600  0.55667
## TissueSalivary_gland       0.4782     0.4657   1.027  0.31983
## TissueFat_bodies           0.6835     0.4657   1.468  0.16161
## TissueTestes               1.4202     0.4657   3.049  0.00765 **
## TissueMid_gut              1.2264     0.4657   2.633  0.01807 *
## TissueBrain                0.8469     0.4657   1.818  0.08777 .
## TissueMalpighian_tubules   0.6252     0.4657   1.342  0.19822
## ---
## Signif. codes:  0 '***' 0.001 '**' 0.01 '*' 0.05 '.' 0.1 ' ' 1
##
## (Dispersion parameter for gaussian family taken to be 0.3253694)
##
##     Null deviance: 9.8230  on 23  degrees of freedom
## Residual deviance: 5.2059  on 16  degrees of freedom
## AIC: 49.431
##
## Number of Fisher Scoring iterations: 2
```

```
Anova(model1)
```

```
## Analysis of Deviance Table (Type II tests)
##
## Response: Normalized
##        LR Chisq Df Pr(>Chisq)
## Tissue    14.19  7     0.0479 *
## ---
## Signif. codes:  0 '***' 0.001 '**' 0.01 '*' 0.05 '.' 0.1 ' ' 1
```

```
fig1iflam$Tissue <- relevel(fig1iflam$Tissue, ref=
"Malpighian_tubules")
model1<-glm(Normalized ~ Tissue, data = fig1iflam)
summary(model1)
```

```
##
## Call:
## glm(formula = Normalized ~ Tissue, data = fig1iflam)
##
## Deviance Residuals:
##     Min       1Q   Median       3Q      Max
## -1.0449  -0.3018  -0.0063   0.1800   1.4493
##
## Coefficients:
##                      Estimate Std. Error t value Pr(>|t|)
## (Intercept)           0.80934    0.32933   2.458   0.0258 *
```

```
## TissueHind_gut       -0.62519    0.46574  -1.342   0.1982
## TissueFront_gut      -0.34557    0.46574  -0.742   0.4688
## TissueSalivary_gland -0.14701    0.46574  -0.316   0.7564
## TissueFat_bodies      0.05831    0.46574   0.125   0.9019
## TissueTestes          0.79503    0.46574   1.707   0.1071
## TissueMid_gut         0.60124    0.46574   1.291   0.2151
## TissueBrain           0.22172    0.46574   0.476   0.6405
## ---
## Signif. codes:  0 '***' 0.001 '**' 0.01 '*' 0.05 '.' 0.1 ' ' 1
##
## (Dispersion parameter for gaussian family taken to be 0.3253694)
##
##     Null deviance: 9.8230  on 23  degrees of freedom
## Residual deviance: 5.2059  on 16  degrees of freedom
## AIC: 49.431
##
## Number of Fisher Scoring iterations: 2

Anova(model1)

## Analysis of Deviance Table (Type II tests)
##
## Response: Normalized
##        LR Chisq Df Pr(>Chisq)
## Tissue    14.19  7     0.0479 *
## ---
## Signif. codes:  0 '***' 0.001 '**' 0.01 '*' 0.05 '.' 0.1 ' ' 1

#------------------------------------------------------
#work with Iflavirus females

fig1iflaf <- subset(fig1ifla, Sex=="Female")
fig1iflaf

##     Target Sample             Tissue Replicate     Virus
Normalized    Sex
## 8      Ifla     7A         Fat_bodies         1 Iflavirus
10.648584190 Female
## 9      Ifla     7A         Fat_bodies         3 Iflavirus
9.314457044 Female
## 11     Ifla     7A         Fat_bodies         2 Iflavirus
5.013356605 Female
## 13     Ifla     3A Malpighian_tubules         1 Iflavirus
3.923198149 Female
## 18     Ifla     8A              Brain         1 Iflavirus
2.764745961 Female
## 20     Ifla     1A     Salivary_gland         1 Iflavirus
1.918755308 Female
## 21     Ifla     4A            Mid_gut         3 Iflavirus
1.825265012 Female
## 23     Ifla     8A              Brain         2 Iflavirus
```

```
1.786912992 Female
## 27     Ifla      3A Malpighian_tubules          3 Iflavirus
1.411609258 Female
## 28     Ifla      9A         Milk_glands          2 Iflavirus
1.385602181 Female
## 32     Ifla      4A             Mid_gut          1 Iflavirus
1.211337107 Female
## 35     Ifla      9A         Milk_glands          3 Iflavirus
1.178765779 Female
## 39     Ifla      8A               Brain          3 Iflavirus
1.023812750 Female
## 40     Ifla      9A         Milk_glands          1 Iflavirus
1.021891196 Female
## 45     Ifla      1A      Salivary_gland          3 Iflavirus
0.967271882 Female
## 50     Ifla      1A      Salivary_gland          2 Iflavirus
0.817396209 Female
## 54     Ifla      4A             Mid_gut          2 Iflavirus
0.719587999 Female
## 59     Ifla      5A           Front_gut          3 Iflavirus
0.474846292 Female
## 61     Ifla      5A           Front_gut          1 Iflavirus
0.428891911 Female
## 68     Ifla      2A            Hind_gut          1 Iflavirus
0.355361037 Female
## 69     Ifla      3A Malpighian_tubules          2 Iflavirus
0.354209947 Female
## 80     Ifla      2A            Hind_gut          2 Iflavirus
0.146907611 Female
## 81     Ifla      2A            Hind_gut          3 Iflavirus
0.130858443 Female
## 87     Ifla      5A           Front_gut          2 Iflavirus
0.099571356 Female
## 97     Ifla      6A               Ovary          3 Iflavirus
0.021062121 Female
## 98     Ifla      6A               Ovary          1 Iflavirus
0.017170381 Female
## 101    Ifla      6A               Ovary          2 Iflavirus
0.007298148 Female
```

```
fig1iflaf$Tissue <- as.factor(fig1iflaf$Tissue)
fig1iflaf$Tissue <- relevel(fig1iflaf$Tissue, ref= "Brain")
model1<-glm(Normalized ~ Tissue, data = fig1iflaf)
summary(model1)
```

```
##
## Call:
## glm(formula = Normalized ~ Tissue, data = fig1iflaf)
##
## Deviance Residuals:
```

```
##      Min       1Q   Median       3Q      Max  
## -3.3121  -0.2510  -0.0167   0.1673   2.3231  
## 
## Coefficients:
##                           Estimate Std. Error t value Pr(>|t|)    
## (Intercept)                1.85849    0.70840   2.624   0.0172 *  
## TissueFat_bodies           6.46698    1.00182   6.455  4.5e-06 ***
## TissueFront_gut           -1.52405    1.00182  -1.521   0.1456    
## TissueHind_gut            -1.64745    1.00182  -1.644   0.1174    
## TissueMalpighian_tubules   0.03785    1.00182   0.038   0.9703    
## TissueMid_gut             -0.60643    1.00182  -0.605   0.5525    
## TissueMilk_glands         -0.66307    1.00182  -0.662   0.5164    
## TissueOvary               -1.84331    1.00182  -1.840   0.0823 .  
## TissueSalivary_gland      -0.62402    1.00182  -0.623   0.5412    
## ---
## Signif. codes:  0 '***' 0.001 '**' 0.01 '*' 0.05 '.' 0.1 ' ' 1
## 
## (Dispersion parameter for gaussian family taken to be 1.505478)
## 
##     Null deviance: 181.408  on 26  degrees of freedom
## Residual deviance:  27.099  on 18  degrees of freedom
## AIC: 96.721
## 
## Number of Fisher Scoring iterations: 2
```

```
Anova(model1)
```

```
## Analysis of Deviance Table (Type II tests)
## 
## Response: Normalized
##        LR Chisq Df Pr(>Chisq)    
## Tissue    102.5  8  < 2.2e-16 ***
## ---
## Signif. codes:  0 '***' 0.001 '**' 0.01 '*' 0.05 '.' 0.1 ' ' 1
```

```
fig1iflaf$Tissue <- relevel(fig1iflaf$Tissue, ref= "Mid_gut")
model1<-glm(Normalized ~ Tissue, data = fig1iflaf)
summary(model1)
```

```
## 
## Call:
## glm(formula = Normalized ~ Tissue, data = fig1iflaf)
## 
## Deviance Residuals: 
##      Min       1Q   Median       3Q      Max  
## -3.3121  -0.2510  -0.0167   0.1673   2.3231  
## 
## Coefficients:
##                           Estimate Std. Error t value Pr(>|t|)  
## (Intercept)                1.25206    0.70840   1.767   0.0941 .
## TissueBrain                0.60643    1.00182   0.605   0.5525  
```

```
## TissueFat_bodies          7.07340    1.00182   7.061 1.38e-06 ***
## TissueFront_gut          -0.91763    1.00182  -0.916   0.3718
## TissueHind_gut           -1.04102    1.00182  -1.039   0.3125
## TissueMalpighian_tubules  0.64428    1.00182   0.643   0.5283
## TissueMilk_glands        -0.05664    1.00182  -0.057   0.9555
## TissueOvary              -1.23689    1.00182  -1.235   0.2328
## TissueSalivary_gland     -0.01759    1.00182  -0.018   0.9862
## ---
## Signif. codes:  0 '***' 0.001 '**' 0.01 '*' 0.05 '.' 0.1 ' ' 1
##
## (Dispersion parameter for gaussian family taken to be 1.505478)
##
##     Null deviance: 181.408  on 26  degrees of freedom
## Residual deviance:  27.099  on 18  degrees of freedom
## AIC: 96.721
##
## Number of Fisher Scoring iterations: 2
```

```
Anova(model1)
```

```
## Analysis of Deviance Table (Type II tests)
##
## Response: Normalized
##        LR Chisq Df Pr(>Chisq)
## Tissue    102.5  8  < 2.2e-16 ***
## ---
## Signif. codes:  0 '***' 0.001 '**' 0.01 '*' 0.05 '.' 0.1 ' ' 1
```

```
fig1iflaf$Tissue <- relevel(fig1iflaf$Tissue, ref= "Ovary")
model1<-glm(Normalized ~ Tissue, data = fig1iflaf)
summary(model1)
```

```
##
## Call:
## glm(formula = Normalized ~ Tissue, data = fig1iflaf)
##
## Deviance Residuals:
##     Min       1Q   Median       3Q      Max
## -3.3121  -0.2510  -0.0167   0.1673   2.3231
##
## Coefficients:
##                          Estimate Std. Error t value Pr(>|t|)
## (Intercept)               0.01518    0.70840   0.021   0.9831
## TissueMid_gut             1.23689    1.00182   1.235   0.2328
## TissueBrain               1.84331    1.00182   1.840   0.0823 .
## TissueFat_bodies          8.31029    1.00182   8.295 1.46e-07 ***
## TissueFront_gut           0.31926    1.00182   0.319   0.7536
## TissueHind_gut            0.19587    1.00182   0.196   0.8472
## TissueMalpighian_tubules  1.88116    1.00182   1.878   0.0767 .
## TissueMilk_glands         1.18024    1.00182   1.178   0.2541
## TissueSalivary_gland      1.21930    1.00182   1.217   0.2393
```

```
## ---
## Signif. codes:  0 '***' 0.001 '**' 0.01 '*' 0.05 '.' 0.1 ' ' 1
##
## (Dispersion parameter for gaussian family taken to be 1.505478)
##
##     Null deviance: 181.408  on 26  degrees of freedom
## Residual deviance:  27.099  on 18  degrees of freedom
## AIC: 96.721
##
## Number of Fisher Scoring iterations: 2
```

```
Anova(model1)
```

```
## Analysis of Deviance Table (Type II tests)
##
## Response: Normalized
##        LR Chisq Df Pr(>Chisq)
## Tissue    102.5  8  < 2.2e-16 ***
## ---
## Signif. codes:  0 '***' 0.001 '**' 0.01 '*' 0.05 '.' 0.1 ' ' 1
```

```
fig1iflaf$Tissue <- relevel(fig1iflaf$Tissue, ref= "Fat_bodies")
model1<-glm(Normalized ~ Tissue, data = fig1iflaf)
summary(model1)
```

```
##
## Call:
## glm(formula = Normalized ~ Tissue, data = fig1iflaf)
##
## Deviance Residuals:
##     Min       1Q   Median       3Q      Max
## -3.3121  -0.2510  -0.0167   0.1673   2.3231
##
## Coefficients:
##                             Estimate Std. Error t value Pr(>|t|)
## (Intercept)                   8.3255     0.7084  11.753 7.05e-10 ***
## TissueOvary                  -8.3103     1.0018  -8.295 1.46e-07 ***
## TissueMid_gut                -7.0734     1.0018  -7.061 1.38e-06 ***
## TissueBrain                  -6.4670     1.0018  -6.455 4.50e-06 ***
## TissueFront_gut              -7.9910     1.0018  -7.976 2.56e-07 ***
## TissueHind_gut               -8.1144     1.0018  -8.100 2.05e-07 ***
## TissueMalpighian_tubules     -6.4291     1.0018  -6.417 4.85e-06 ***
## TissueMilk_glands            -7.1300     1.0018  -7.117 1.24e-06 ***
## TissueSalivary_gland         -7.0910     1.0018  -7.078 1.34e-06 ***
## ---
## Signif. codes:  0 '***' 0.001 '**' 0.01 '*' 0.05 '.' 0.1 ' ' 1
##
## (Dispersion parameter for gaussian family taken to be 1.505478)
##
##     Null deviance: 181.408  on 26  degrees of freedom
## Residual deviance:  27.099  on 18  degrees of freedom
```

```
## AIC: 96.721
##
## Number of Fisher Scoring iterations: 2
```

```
Anova(model1)
```

```
## Analysis of Deviance Table (Type II tests)
##
## Response: Normalized
##        LR Chisq Df Pr(>Chisq)
## Tissue    102.5  8  < 2.2e-16 ***
## ---
## Signif. codes:  0 '***' 0.001 '**' 0.01 '*' 0.05 '.' 0.1 ' ' 1
```

```
fig1iflaf$Tissue <- relevel(fig1iflaf$Tissue, ref= "Salivary_gland")
model1<-glm(Normalized ~ Tissue, data = fig1iflaf)
summary(model1)
```

```
##
## Call:
## glm(formula = Normalized ~ Tissue, data = fig1iflaf)
##
## Deviance Residuals:
##     Min       1Q   Median       3Q      Max
## -3.3121  -0.2510  -0.0167   0.1673   2.3231
##
## Coefficients:
##                            Estimate Std. Error t value Pr(>|t|)
## (Intercept)                 1.23447    0.70840   1.743   0.0985 .
## TissueFat_bodies            7.09099    1.00182   7.078 1.34e-06 ***
## TissueOvary                -1.21930    1.00182  -1.217   0.2393
## TissueMid_gut               0.01759    1.00182   0.018   0.9862
## TissueBrain                 0.62402    1.00182   0.623   0.5412
## TissueFront_gut            -0.90004    1.00182  -0.898   0.3808
## TissueHind_gut             -1.02343    1.00182  -1.022   0.3205
## TissueMalpighian_tubules    0.66186    1.00182   0.661   0.5172
## TissueMilk_glands          -0.03905    1.00182  -0.039   0.9693
## ---
## Signif. codes:  0 '***' 0.001 '**' 0.01 '*' 0.05 '.' 0.1 ' ' 1
##
## (Dispersion parameter for gaussian family taken to be 1.505478)
##
##     Null deviance: 181.408  on 26  degrees of freedom
## Residual deviance:  27.099  on 18  degrees of freedom
## AIC: 96.721
##
## Number of Fisher Scoring iterations: 2
```

```
Anova(model1)
```

```
## Analysis of Deviance Table (Type II tests)
##
## Response: Normalized
##        LR Chisq Df Pr(>Chisq)
## Tissue    102.5  8  < 2.2e-16 ***
## ---
## Signif. codes:  0 '***' 0.001 '**' 0.01 '*' 0.05 '.' 0.1 ' ' 1
```

```
fig1iflaf$Tissue <- relevel(fig1iflaf$Tissue, ref= "Front_gut")
model1<-glm(Normalized ~ Tissue, data = fig1iflaf)
summary(model1)
```

```
##
## Call:
## glm(formula = Normalized ~ Tissue, data = fig1iflaf)
##
## Deviance Residuals:
##     Min       1Q   Median       3Q      Max
## -3.3121  -0.2510  -0.0167   0.1673   2.3231
##
## Coefficients:
##                            Estimate Std. Error t value Pr(>|t|)
## (Intercept)                  0.3344     0.7084   0.472    0.643
## TissueSalivary_gland         0.9000     1.0018   0.898    0.381
## TissueFat_bodies             7.9910     1.0018   7.976 2.56e-07 ***
## TissueOvary                 -0.3193     1.0018  -0.319    0.754
## TissueMid_gut                0.9176     1.0018   0.916    0.372
## TissueBrain                  1.5241     1.0018   1.521    0.146
## TissueHind_gut              -0.1234     1.0018  -0.123    0.903
## TissueMalpighian_tubules     1.5619     1.0018   1.559    0.136
## TissueMilk_glands            0.8610     1.0018   0.859    0.401
## ---
## Signif. codes:  0 '***' 0.001 '**' 0.01 '*' 0.05 '.' 0.1 ' ' 1
##
## (Dispersion parameter for gaussian family taken to be 1.505478)
##
##     Null deviance: 181.408  on 26  degrees of freedom
## Residual deviance:  27.099  on 18  degrees of freedom
## AIC: 96.721
##
## Number of Fisher Scoring iterations: 2
```

```
Anova(model1)
```

```
## Analysis of Deviance Table (Type II tests)
##
## Response: Normalized
##        LR Chisq Df Pr(>Chisq)
## Tissue    102.5  8  < 2.2e-16 ***
## ---
## Signif. codes:  0 '***' 0.001 '**' 0.01 '*' 0.05 '.' 0.1 ' ' 1
```

```
fig1iflaf$Tissue <- relevel(fig1iflaf$Tissue, ref= "Hind_gut")
model1<-glm(Normalized ~ Tissue, data = fig1iflaf)
summary(model1)

##
## Call:
## glm(formula = Normalized ~ Tissue, data = fig1iflaf)
##
## Deviance Residuals:
##     Min       1Q   Median       3Q      Max
## -3.3121  -0.2510  -0.0167   0.1673   2.3231
##
## Coefficients:
##                            Estimate Std. Error t value Pr(>|t|)
## (Intercept)                  0.2110     0.7084   0.298    0.769
## TissueFront_gut              0.1234     1.0018   0.123    0.903
## TissueSalivary_gland         1.0234     1.0018   1.022    0.321
## TissueFat_bodies             8.1144     1.0018   8.100 2.05e-07 ***
## TissueOvary                 -0.1959     1.0018  -0.196    0.847
## TissueMid_gut                1.0410     1.0018   1.039    0.313
## TissueBrain                  1.6474     1.0018   1.644    0.117
## TissueMalpighian_tubules     1.6853     1.0018   1.682    0.110
## TissueMilk_glands            0.9844     1.0018   0.983    0.339
## ---
## Signif. codes:  0 '***' 0.001 '**' 0.01 '*' 0.05 '.' 0.1 ' ' 1
##
## (Dispersion parameter for gaussian family taken to be 1.505478)
##
##     Null deviance: 181.408  on 26  degrees of freedom
## Residual deviance:  27.099  on 18  degrees of freedom
## AIC: 96.721
##
## Number of Fisher Scoring iterations: 2

Anova(model1)

## Analysis of Deviance Table (Type II tests)
##
## Response: Normalized
##        LR Chisq Df Pr(>Chisq)
## Tissue    102.5  8  < 2.2e-16 ***
## ---
## Signif. codes:  0 '***' 0.001 '**' 0.01 '*' 0.05 '.' 0.1 ' ' 1

fig1iflaf$Tissue <- relevel(fig1iflaf$Tissue, ref= "Milk_glands")
model1<-glm(Normalized ~ Tissue, data = fig1iflaf)
summary(model1)

##
## Call:
## glm(formula = Normalized ~ Tissue, data = fig1iflaf)
```

```
##
## Deviance Residuals:
##     Min       1Q   Median       3Q      Max
## -3.3121  -0.2510  -0.0167   0.1673   2.3231
##
## Coefficients:
##                            Estimate Std. Error t value Pr(>|t|)
## (Intercept)                 1.19542    0.70840   1.687    0.109
## TissueHind_gut             -0.98438    1.00182  -0.983    0.339
## TissueFront_gut            -0.86098    1.00182  -0.859    0.401
## TissueSalivary_gland        0.03905    1.00182   0.039    0.969
## TissueFat_bodies            7.13005    1.00182   7.117 1.24e-06 ***
## TissueOvary                -1.18024    1.00182  -1.178    0.254
## TissueMid_gut               0.05664    1.00182   0.057    0.956
## TissueBrain                 0.66307    1.00182   0.662    0.516
## TissueMalpighian_tubules  0.70092    1.00182   0.700    0.493
## ---
## Signif. codes:  0 '***' 0.001 '**' 0.01 '*' 0.05 '.' 0.1 ' ' 1
##
## (Dispersion parameter for gaussian family taken to be 1.505478)
##
##     Null deviance: 181.408  on 26  degrees of freedom
## Residual deviance:  27.099  on 18  degrees of freedom
## AIC: 96.721
##
## Number of Fisher Scoring iterations: 2
```

```
Anova(model1)
```

```
## Analysis of Deviance Table (Type II tests)
##
## Response: Normalized
##        LR Chisq Df Pr(>Chisq)
## Tissue    102.5  8  < 2.2e-16 ***
## ---
## Signif. codes:  0 '***' 0.001 '**' 0.01 '*' 0.05 '.' 0.1 ' ' 1
```

```
fig1iflaf$Tissue <- relevel(fig1iflaf$Tissue, ref=
"Malpighian_tubules")
model1<-glm(Normalized ~ Tissue, data = fig1iflaf)
summary(model1)
```

```
##
## Call:
## glm(formula = Normalized ~ Tissue, data = fig1iflaf)
##
## Deviance Residuals:
##     Min       1Q   Median       3Q      Max
## -3.3121  -0.2510  -0.0167   0.1673   2.3231
##
## Coefficients:
```

```
##                        Estimate Std. Error t value Pr(>|t|)
## (Intercept)             1.89634    0.70840   2.677   0.0154 *
## TissueMilk_glands      -0.70092    1.00182  -0.700   0.4931
## TissueHind_gut         -1.68530    1.00182  -1.682   0.1098
## TissueFront_gut        -1.56190    1.00182  -1.559   0.1364
## TissueSalivary_gland -0.66186    1.00182  -0.661   0.5172
## TissueFat_bodies        6.42913    1.00182   6.417 4.85e-06 ***
## TissueOvary            -1.88116    1.00182  -1.878   0.0767 .
## TissueMid_gut          -0.64428    1.00182  -0.643   0.5283
## TissueBrain            -0.03785    1.00182  -0.038   0.9703
## ---
## Signif. codes:  0 '***' 0.001 '**' 0.01 '*' 0.05 '.' 0.1 ' ' 1
##
## (Dispersion parameter for gaussian family taken to be 1.505478)
##
##     Null deviance: 181.408  on 26  degrees of freedom
## Residual deviance:  27.099  on 18  degrees of freedom
## AIC: 96.721
##
## Number of Fisher Scoring iterations: 2
```

```
Anova(model1)
```

```
## Analysis of Deviance Table (Type II tests)
##
## Response: Normalized
##        LR Chisq Df Pr(>Chisq)
## Tissue    102.5  8  < 2.2e-16 ***
## ---
## Signif. codes:  0 '***' 0.001 '**' 0.01 '*' 0.05 '.' 0.1 ' ' 1
```

statistical analysis for Negevirus

```
#work for Negevirus

fig1nege <- subset(fig1, Virus=="Negevirus")
fig1nege
```

```
##     Target Sample             Tissue Replicate     Virus
Normalized    Sex
## 1      Nege     1A     Salivary_gland         2 Negevirus
77.402705950 Female
## 2      Nege     7A         Fat_bodies         3 Negevirus
39.906292910   Male
## 3      Nege     7A         Fat_bodies         1 Negevirus
39.349594060 Female
## 4      Nege     1A     Salivary_gland         3 Negevirus
29.069334440 Female
## 5      Nege     7A         Fat_bodies         1 Negevirus
20.357023310   Male
## 6      Nege     7A         Fat_bodies         2 Negevirus
```

```
19.631228500 Female
## 7     Nege     1A     Salivary_gland         2 Negevirus
18.722890740   Male
## 10    Nege     3A Malpighian_tubules         1 Negevirus
7.597307635 Female
## 12    Nege     1A     Salivary_gland         1 Negevirus
4.376742078   Male
## 14    Nege     7A         Fat_bodies         2 Negevirus
3.537905209   Male
## 15    Nege     7A         Fat_bodies         3 Negevirus
3.331728226 Female
## 16    Nege     1A     Salivary_gland         3 Negevirus
2.908747434   Male
## 19    Nege     8A              Brain         1 Negevirus
2.665310756 Female
## 24    Nege     8A              Brain         2 Negevirus
1.773831321 Female
## 26    Nege     9A        Milk_glands         2 Negevirus
1.412966980 Female
## 36    Nege     5A          Front_gut         1 Negevirus
1.141186814 Female
## 37    Nege     8A              Brain         1 Negevirus
1.060610153   Male
## 38    Nege     6A            Testes          3 Negevirus
1.048261614   Male
## 43    Nege     6A              Ovary         1 Negevirus
0.996945359 Female
## 44    Nege     6A            Testes          1 Negevirus
0.981968623   Male
## 46    Nege     4A            Mid_gut         3 Negevirus
0.944433541   Male
## 49    Nege     5A          Front_gut         3 Negevirus
0.852571665   Male
## 53    Nege     4A            Mid_gut         1 Negevirus
0.751201684 Female
## 56    Nege     2A           Hind_gut         1 Negevirus
0.596460970 Female
## 57    Nege     9A        Milk_glands         1 Negevirus
0.579908913 Female
## 60    Nege     8A              Brain         3 Negevirus
0.454187308   Male
## 62    Nege     4A            Mid_gut         1 Negevirus
0.417650378   Male
## 65    Nege     9A        Milk_glands         3 Negevirus
0.371587771 Female
## 67    Nege     1A     Salivary_gland         1 Negevirus
0.358316060 Female
## 70    Nege     6A            Testes          2 Negevirus
0.352838330   Male
## 71    Nege     6A              Ovary         2 Negevirus
```

```
0.352644646 Female
## 72     Nege      4A             Mid_gut          2 Negevirus
0.321005816 Female
## 74     Nege      2A            Hind_gut          1 Negevirus
0.246349379   Male
## 75     Nege      3A Malpighian_tubules          3 Negevirus
0.225166941   Male
## 76     Nege      5A           Front_gut          1 Negevirus
0.196212798   Male
## 77     Nege      8A               Brain          2 Negevirus
0.179375639   Male
## 79     Nege      3A Malpighian_tubules          2 Negevirus
0.159920548 Female
## 85     Nege      3A Malpighian_tubules          1 Negevirus
0.103543820   Male
## 86     Nege      5A           Front_gut          2 Negevirus
0.100733703 Female
## 88     Nege      4A             Mid_gut          3 Negevirus
0.094092464 Female
## 89     Nege      2A            Hind_gut          2 Negevirus
0.089570912 Female
## 90     Nege      5A           Front_gut          2 Negevirus
0.071801559   Male
## 91     Nege      8A               Brain          3 Negevirus
0.057932219 Female
## 92     Nege      3A Malpighian_tubules          3 Negevirus
0.042103969 Female
## 93     Nege      5A           Front_gut          3 Negevirus
0.039952549 Female
## 94     Nege      6A               Ovary          3 Negevirus
0.032297897 Female
## 95     Nege      4A             Mid_gut          2 Negevirus
0.028138818   Male
## 96     Nege      2A            Hind_gut          3 Negevirus
0.023073452   Male
## 99     Nege      3A Malpighian_tubules          2 Negevirus
0.015372781   Male
## 100    Nege      2A            Hind_gut          3 Negevirus
0.012167280 Female
## 102    Nege      2A            Hind_gut          2 Negevirus
0.003874746   Male

#difference between tissue regardsless the sex for Negevirus
model1<-glm(Normalized ~ Tissue, data = fig1nege)
summary(model1)

##
## Call:
## glm(formula = Normalized ~ Tissue, data = fig1nege)
##
```

```
## Deviance Residuals:
##     Min       1Q   Median       3Q      Max
## -21.781   -0.913   -0.204    0.380   55.263
##
## Coefficients:
##                            Estimate Std. Error t value Pr(>|t|)
## (Intercept)                  1.0319     4.7892   0.215  0.83048
## TissueFat_bodies            19.9871     6.7730   2.951  0.00522 **
## TissueFront_gut             -0.6315     6.7730  -0.093  0.92617
## TissueHind_gut              -0.8700     6.7730  -0.128  0.89842
## TissueMalpighian_tubules     0.3254     6.7730   0.048  0.96192
## TissueMid_gut               -0.6058     6.7730  -0.089  0.92917
## TissueMilk_glands           -0.2437     8.2952  -0.029  0.97670
## TissueOvary                 -0.5712     8.2952  -0.069  0.94543
## TissueSalivary_gland        21.1079     6.7730   3.116  0.00334 **
## TissueTestes                -0.2375     8.2952  -0.029  0.97730
## ---
## Signif. codes:  0 '***' 0.001 '**' 0.01 '*' 0.05 '.' 0.1 ' ' 1
##
## (Dispersion parameter for gaussian family taken to be 137.62)
##
##     Null deviance: 9661.7  on 50  degrees of freedom
## Residual deviance: 5642.4  on 41  degrees of freedom
## AIC: 406.75
##
## Number of Fisher Scoring iterations: 2
```

```
Anova(model1)
```

```
## Analysis of Deviance Table (Type II tests)
##
## Response: Normalized
##        LR Chisq Df Pr(>Chisq)
## Tissue   29.206  9  0.0005982 ***
## ---
## Signif. codes:  0 '***' 0.001 '**' 0.01 '*' 0.05 '.' 0.1 ' ' 1
```

```
#difference between sex regardsless the tissues for Negevirus
model1<-glm(Normalized ~ Tissue, data = fig1nege)
summary(model1)
```

```
##
## Call:
## glm(formula = Normalized ~ Tissue, data = fig1nege)
##
## Deviance Residuals:
##     Min       1Q   Median       3Q      Max
## -21.781   -0.913   -0.204    0.380   55.263
##
## Coefficients:
##                            Estimate Std. Error t value Pr(>|t|)
```

```
## (Intercept)                 1.0319     4.7892   0.215  0.83048
## TissueFat_bodies           19.9871     6.7730   2.951  0.00522 **
## TissueFront_gut            -0.6315     6.7730  -0.093  0.92617
## TissueHind_gut             -0.8700     6.7730  -0.128  0.89842
## TissueMalpighian_tubules    0.3254     6.7730   0.048  0.96192
## TissueMid_gut              -0.6058     6.7730  -0.089  0.92917
## TissueMilk_glands          -0.2437     8.2952  -0.029  0.97670
## TissueOvary                -0.5712     8.2952  -0.069  0.94543
## TissueSalivary_gland       21.1079     6.7730   3.116  0.00334 **
## TissueTestes               -0.2375     8.2952  -0.029  0.97730
## ---
## Signif. codes:  0 '***' 0.001 '**' 0.01 '*' 0.05 '.' 0.1 ' ' 1
##
## (Dispersion parameter for gaussian family taken to be 137.62)
##
##     Null deviance: 9661.7  on 50  degrees of freedom
## Residual deviance: 5642.4  on 41  degrees of freedom
## AIC: 406.75
##
## Number of Fisher Scoring iterations: 2
```

```
Anova(model1)
```

```
## Analysis of Deviance Table (Type II tests)
##
## Response: Normalized
##        LR Chisq Df Pr(>Chisq)
## Tissue   29.206  9  0.0005982 ***
## ---
## Signif. codes:  0 '***' 0.001 '**' 0.01 '*' 0.05 '.' 0.1 ' ' 1
```

```
#for male
fig1negem <- subset(fig1nege, Sex=="Male")
fig1negem
```

```
##     Target Sample            Tissue Replicate     Virus
Normalized  Sex
## 2      Nege     7A        Fat_bodies         3 Negevirus
39.906292910 Male
## 5      Nege     7A        Fat_bodies         1 Negevirus
20.357023310 Male
## 7      Nege     1A    Salivary_gland         2 Negevirus
18.722890740 Male
## 12     Nege     1A    Salivary_gland         1 Negevirus
4.376742078 Male
## 14     Nege     7A        Fat_bodies         2 Negevirus
3.537905209 Male
## 16     Nege     1A    Salivary_gland         3 Negevirus
2.908747434 Male
## 37     Nege     8A             Brain         1 Negevirus
1.060610153 Male
```

```
## 38     Nege      6A             Testes         3 Negevirus
1.048261614 Male
## 44     Nege      6A             Testes         1 Negevirus
0.981968623 Male
## 46     Nege      4A            Mid_gut         3 Negevirus
0.944433541 Male
## 49     Nege      5A          Front_gut         3 Negevirus
0.852571665 Male
## 60     Nege      8A              Brain         3 Negevirus
0.454187308 Male
## 62     Nege      4A            Mid_gut         1 Negevirus
0.417650378 Male
## 70     Nege      6A             Testes         2 Negevirus
0.352838330 Male
## 74     Nege      2A           Hind_gut         1 Negevirus
0.246349379 Male
## 75     Nege      3A Malpighian_tubules         3 Negevirus
0.225166941 Male
## 76     Nege      5A          Front_gut         1 Negevirus
0.196212798 Male
## 77     Nege      8A              Brain         2 Negevirus
0.179375639 Male
## 85     Nege      3A Malpighian_tubules         1 Negevirus
0.103543820 Male
## 90     Nege      5A          Front_gut         2 Negevirus
0.071801559 Male
## 95     Nege      4A            Mid_gut         2 Negevirus
0.028138818 Male
## 96     Nege      2A           Hind_gut         3 Negevirus
0.023073452 Male
## 99     Nege      3A Malpighian_tubules         2 Negevirus
0.015372781 Male
## 102    Nege      2A           Hind_gut         2 Negevirus
0.003874746 Male
```

```
fig1negem$Tissue <- as.factor(fig1negem$Tissue)
fig1negem$Tissue <- relevel(fig1negem$Tissue, ref= "Brain")
model1<-glm(Normalized ~ Tissue, data = fig1negem)
summary(model1)
```

```
##
## Call:
## glm(formula = Normalized ~ Tissue, data = fig1negem)
##
## Deviance Residuals:
##      Min        1Q    Median        3Q       Max
## -17.7292   -0.3978   -0.0776    0.2042   18.6392
##
## Coefficients:
##                           Estimate Std. Error t value Pr(>|t|)
```

```
## (Intercept)                 0.5647     4.1251   0.137  0.89282
## TissueFat_bodies           20.7023     5.8338   3.549  0.00267 **
## TissueFront_gut            -0.1912     5.8338  -0.033  0.97426
## TissueHind_gut             -0.4736     5.8338  -0.081  0.93630
## TissueMalpighian_tubules   -0.4500     5.8338  -0.077  0.93947
## TissueMid_gut              -0.1013     5.8338  -0.017  0.98636
## TissueSalivary_gland        8.1047     5.8338   1.389  0.18378
## TissueTestes                0.2296     5.8338   0.039  0.96909
## ---
## Signif. codes:  0 '***' 0.001 '**' 0.01 '*' 0.05 '.' 0.1 ' ' 1
##
## (Dispersion parameter for gaussian family taken to be 51.04947)
##
##     Null deviance: 2010.95  on 23  degrees of freedom
## Residual deviance:  816.79  on 16  degrees of freedom
## AIC: 170.76
##
## Number of Fisher Scoring iterations: 2
```

```
Anova(model1)
```

```
## Analysis of Deviance Table (Type II tests)
##
## Response: Normalized
##        LR Chisq Df Pr(>Chisq)
## Tissue   23.392  7   0.001456 **
## ---
## Signif. codes:  0 '***' 0.001 '**' 0.01 '*' 0.05 '.' 0.1 ' ' 1
```

```
fig1negem$Tissue <- relevel(fig1negem$Tissue, ref= "Mid_gut")
model1<-glm(Normalized ~ Tissue, data = fig1negem)
summary(model1)
```

```
##
## Call:
## glm(formula = Normalized ~ Tissue, data = fig1negem)
##
## Deviance Residuals:
##      Min        1Q    Median        3Q       Max
## -17.7292   -0.3978   -0.0776    0.2042   18.6392
##
## Coefficients:
##                          Estimate Std. Error t value Pr(>|t|)
## (Intercept)               0.46341    4.12510   0.112  0.91195
## TissueBrain               0.10132    5.83378   0.017  0.98636
## TissueFat_bodies         20.80367    5.83378   3.566  0.00258 **
## TissueFront_gut          -0.08988    5.83378  -0.015  0.98790
## TissueHind_gut           -0.37231    5.83378  -0.064  0.94990
## TissueMalpighian_tubules -0.34871    5.83378  -0.060  0.95308
## TissueSalivary_gland      8.20605    5.83378   1.407  0.17866
## TissueTestes              0.33095    5.83378   0.057  0.95546
```

```
## ---
## Signif. codes:  0 '***' 0.001 '**' 0.01 '*' 0.05 '.' 0.1 ' ' 1
##
## (Dispersion parameter for gaussian family taken to be 51.04947)
##
##     Null deviance: 2010.95  on 23  degrees of freedom
## Residual deviance:  816.79  on 16  degrees of freedom
## AIC: 170.76
##
## Number of Fisher Scoring iterations: 2
```

```
Anova(model1)
```

```
## Analysis of Deviance Table (Type II tests)
##
## Response: Normalized
##        LR Chisq Df Pr(>Chisq)
## Tissue   23.392  7   0.001456 **
## ---
## Signif. codes:  0 '***' 0.001 '**' 0.01 '*' 0.05 '.' 0.1 ' ' 1
```

```
fig1negem$Tissue <- relevel(fig1negem$Tissue, ref= "Testes")
model1<-glm(Normalized ~ Tissue, data = fig1negem)
summary(model1)
```

```
##
## Call:
## glm(formula = Normalized ~ Tissue, data = fig1negem)
##
## Deviance Residuals:
##      Min        1Q    Median        3Q       Max
## -17.7292   -0.3978   -0.0776    0.2042   18.6392
##
## Coefficients:
##                            Estimate Std. Error t value Pr(>|t|)
## (Intercept)                  0.7944     4.1251   0.193  0.84972
## TissueMid_gut               -0.3309     5.8338  -0.057  0.95546
## TissueBrain                 -0.2296     5.8338  -0.039  0.96909
## TissueFat_bodies            20.4727     5.8338   3.509  0.00291 **
## TissueFront_gut             -0.4208     5.8338  -0.072  0.94339
## TissueHind_gut              -0.7033     5.8338  -0.121  0.90555
## TissueMalpighian_tubules   -0.6797     5.8338  -0.117  0.90870
## TissueSalivary_gland         7.8751     5.8338   1.350  0.19583
## ---
## Signif. codes:  0 '***' 0.001 '**' 0.01 '*' 0.05 '.' 0.1 ' ' 1
##
## (Dispersion parameter for gaussian family taken to be 51.04947)
##
##     Null deviance: 2010.95  on 23  degrees of freedom
## Residual deviance:  816.79  on 16  degrees of freedom
## AIC: 170.76
```

```
##
## Number of Fisher Scoring iterations: 2
```

```
Anova(model1)
```

```
## Analysis of Deviance Table (Type II tests)
##
## Response: Normalized
##        LR Chisq Df Pr(>Chisq)
## Tissue   23.392  7   0.001456 **
## ---
## Signif. codes:  0 '***' 0.001 '**' 0.01 '*' 0.05 '.' 0.1 ' ' 1
```

```
fig1negem$Tissue <- relevel(fig1negem$Tissue, ref= "Fat_bodies")
model1<-glm(Normalized ~ Tissue, data = fig1negem)
summary(model1)
```

```
##
## Call:
## glm(formula = Normalized ~ Tissue, data = fig1negem)
##
## Deviance Residuals:
##      Min        1Q    Median        3Q       Max
## -17.7292   -0.3978   -0.0776    0.2042   18.6392
##
## Coefficients:
##                          Estimate Std. Error t value Pr(>|t|)
## (Intercept)                21.267      4.125   5.156 9.58e-05 ***
## TissueTestes              -20.473      5.834  -3.509  0.00291 **
## TissueMid_gut             -20.804      5.834  -3.566  0.00258 **
## TissueBrain               -20.702      5.834  -3.549  0.00267 **
## TissueFront_gut           -20.894      5.834  -3.581  0.00249 **
## TissueHind_gut            -21.176      5.834  -3.630  0.00225 **
## TissueMalpighian_tubules  -21.152      5.834  -3.626  0.00227 **
## TissueSalivary_gland      -12.598      5.834  -2.159  0.04634 *
## ---
## Signif. codes:  0 '***' 0.001 '**' 0.01 '*' 0.05 '.' 0.1 ' ' 1
##
## (Dispersion parameter for gaussian family taken to be 51.04947)
##
##     Null deviance: 2010.95  on 23  degrees of freedom
## Residual deviance:  816.79  on 16  degrees of freedom
## AIC: 170.76
##
## Number of Fisher Scoring iterations: 2
```

```
Anova(model1)
```

```
## Analysis of Deviance Table (Type II tests)
##
## Response: Normalized
```

```
##        LR Chisq Df Pr(>Chisq)
## Tissue   23.392  7   0.001456 **
## ---
## Signif. codes:  0 '***' 0.001 '**' 0.01 '*' 0.05 '.' 0.1 ' ' 1

fig1negem$Tissue <- relevel(fig1negem$Tissue, ref= "Salivary_gland")
model1<-glm(Normalized ~ Tissue, data = fig1negem)
summary(model1)

##
## Call:
## glm(formula = Normalized ~ Tissue, data = fig1negem)
##
## Deviance Residuals:
##      Min        1Q    Median        3Q       Max
## -17.7292   -0.3978   -0.0776    0.2042   18.6392
##
## Coefficients:
##                           Estimate Std. Error t value Pr(>|t|)
## (Intercept)                  8.669      4.125   2.102   0.0518 .
## TissueFat_bodies            12.598      5.834   2.159   0.0463 *
## TissueTestes                -7.875      5.834  -1.350   0.1958
## TissueMid_gut               -8.206      5.834  -1.407   0.1787
## TissueBrain                 -8.105      5.834  -1.389   0.1838
## TissueFront_gut             -8.296      5.834  -1.422   0.1742
## TissueHind_gut              -8.578      5.834  -1.470   0.1608
## TissueMalpighian_tubules    -8.555      5.834  -1.466   0.1619
## ---
## Signif. codes:  0 '***' 0.001 '**' 0.01 '*' 0.05 '.' 0.1 ' ' 1
##
## (Dispersion parameter for gaussian family taken to be 51.04947)
##
##     Null deviance: 2010.95  on 23  degrees of freedom
## Residual deviance:  816.79  on 16  degrees of freedom
## AIC: 170.76
##
## Number of Fisher Scoring iterations: 2

Anova(model1)

## Analysis of Deviance Table (Type II tests)
##
## Response: Normalized
##        LR Chisq Df Pr(>Chisq)
## Tissue   23.392  7   0.001456 **
## ---
## Signif. codes:  0 '***' 0.001 '**' 0.01 '*' 0.05 '.' 0.1 ' ' 1

fig1negem$Tissue <- relevel(fig1negem$Tissue, ref= "Front_gut")
model1<-glm(Normalized ~ Tissue, data = fig1negem)
summary(model1)
```

```
##
## Call:
## glm(formula = Normalized ~ Tissue, data = fig1negem)
##
## Deviance Residuals:
##      Min        1Q    Median        3Q       Max
## -17.7292   -0.3978   -0.0776    0.2042   18.6392
##
## Coefficients:
##                            Estimate Std. Error t value Pr(>|t|)
## (Intercept)                 0.37353    4.12510   0.091  0.92897
## TissueSalivary_gland        8.29593    5.83378   1.422  0.17421
## TissueFat_bodies           20.89355    5.83378   3.581  0.00249 **
## TissueTestes                0.42083    5.83378   0.072  0.94339
## TissueMid_gut               0.08988    5.83378   0.015  0.98790
## TissueBrain                 0.19120    5.83378   0.033  0.97426
## TissueHind_gut             -0.28243    5.83378  -0.048  0.96199
## TissueMalpighian_tubules -0.25883    5.83378  -0.044  0.96516
## ---
## Signif. codes:  0 '***' 0.001 '**' 0.01 '*' 0.05 '.' 0.1 ' ' 1
##
## (Dispersion parameter for gaussian family taken to be 51.04947)
##
##     Null deviance: 2010.95  on 23  degrees of freedom
## Residual deviance:  816.79  on 16  degrees of freedom
## AIC: 170.76
##
## Number of Fisher Scoring iterations: 2
```

```
Anova(model1)
```

```
## Analysis of Deviance Table (Type II tests)
##
## Response: Normalized
##        LR Chisq Df Pr(>Chisq)
## Tissue   23.392  7   0.001456 **
## ---
## Signif. codes:  0 '***' 0.001 '**' 0.01 '*' 0.05 '.' 0.1 ' ' 1
```

```
fig1negem$Tissue <- relevel(fig1negem$Tissue, ref= "Hind_gut")
model1<-glm(Normalized ~ Tissue, data = fig1negem)
summary(model1)
```

```
##
## Call:
## glm(formula = Normalized ~ Tissue, data = fig1negem)
##
## Deviance Residuals:
##      Min        1Q    Median        3Q       Max
## -17.7292   -0.3978   -0.0776    0.2042   18.6392
##
```

```
## Coefficients:
##                           Estimate Std. Error t value Pr(>|t|)
## (Intercept)                 0.0911     4.1251   0.022  0.98265
## TissueFront_gut             0.2824     5.8338   0.048  0.96199
## TissueSalivary_gland        8.5784     5.8338   1.470  0.16083
## TissueFat_bodies           21.1760     5.8338   3.630  0.00225 **
## TissueTestes                0.7033     5.8338   0.121  0.90555
## TissueMid_gut               0.3723     5.8338   0.064  0.94990
## TissueBrain                 0.4736     5.8338   0.081  0.93630
## TissueMalpighian_tubules   0.0236     5.8338   0.004  0.99682
## ---
## Signif. codes:  0 '***' 0.001 '**' 0.01 '*' 0.05 '.' 0.1 ' ' 1
##
## (Dispersion parameter for gaussian family taken to be 51.04947)
##
##     Null deviance: 2010.95  on 23  degrees of freedom
## Residual deviance:  816.79  on 16  degrees of freedom
## AIC: 170.76
##
## Number of Fisher Scoring iterations: 2
```

```
Anova(model1)
```

```
## Analysis of Deviance Table (Type II tests)
##
## Response: Normalized
##        LR Chisq Df Pr(>Chisq)
## Tissue   23.392  7   0.001456 **
## ---
## Signif. codes:  0 '***' 0.001 '**' 0.01 '*' 0.05 '.' 0.1 ' ' 1
```

```
fig1negem$Tissue <- relevel(fig1negem$Tissue, ref=
"Malpighian_tubules")
model1<-glm(Normalized ~ Tissue, data = fig1negem)
summary(model1)
```

```
##
## Call:
## glm(formula = Normalized ~ Tissue, data = fig1negem)
##
## Deviance Residuals:
##      Min        1Q    Median        3Q       Max
## -17.7292   -0.3978   -0.0776    0.2042   18.6392
##
## Coefficients:
##                       Estimate Std. Error t value Pr(>|t|)
## (Intercept)             0.1147     4.1251   0.028  0.97816
## TissueHind_gut         -0.0236     5.8338  -0.004  0.99682
## TissueFront_gut         0.2588     5.8338   0.044  0.96516
## TissueSalivary_gland   8.5548     5.8338   1.466  0.16191
## TissueFat_bodies       21.1524     5.8338   3.626  0.00227 **
```

```
## TissueTestes           0.6797     5.8338   0.117  0.90870
## TissueMid_gut          0.3487     5.8338   0.060  0.95308
## TissueBrain            0.4500     5.8338   0.077  0.93947
## ---
## Signif. codes:  0 '***' 0.001 '**' 0.01 '*' 0.05 '.' 0.1 ' ' 1
##
## (Dispersion parameter for gaussian family taken to be 51.04947)
##
##     Null deviance: 2010.95  on 23  degrees of freedom
## Residual deviance:  816.79  on 16  degrees of freedom
## AIC: 170.76
##
## Number of Fisher Scoring iterations: 2
```

```
Anova(model1)
```

```
## Analysis of Deviance Table (Type II tests)
##
## Response: Normalized
##        LR Chisq Df Pr(>Chisq)
## Tissue   23.392  7   0.001456 **
## ---
## Signif. codes:  0 '***' 0.001 '**' 0.01 '*' 0.05 '.' 0.1 ' ' 1
```

```
#------------------------------------------------------
#work with Iflavirus females

fig1negef <- subset(fig1nege, Sex=="Female")
fig1negef
```

```
##     Target Sample            Tissue Replicate     Virus  Normalized
Sex
## 1     Nege     1A     Salivary_gland         2 Negevirus 77.40270595
Female
## 3     Nege     7A         Fat_bodies         1 Negevirus 39.34959406
Female
## 4     Nege     1A     Salivary_gland         3 Negevirus 29.06933444
Female
## 6     Nege     7A         Fat_bodies         2 Negevirus 19.63122850
Female
## 10    Nege     3A Malpighian_tubules         1 Negevirus  7.59730763
Female
## 15    Nege     7A         Fat_bodies         3 Negevirus  3.33172823
Female
## 19    Nege     8A              Brain         1 Negevirus  2.66531076
Female
## 24    Nege     8A              Brain         2 Negevirus  1.77383132
Female
## 26    Nege     9A        Milk_glands         2 Negevirus  1.41296698
Female
## 36    Nege     5A          Front_gut         1 Negevirus  1.14118681
```

```
Female
## 43     Nege      6A                Ovary          1 Negevirus  0.99694536
Female
## 53     Nege      4A              Mid_gut          1 Negevirus  0.75120168
Female
## 56     Nege      2A             Hind_gut          1 Negevirus  0.59646097
Female
## 57     Nege      9A          Milk_glands          1 Negevirus  0.57990891
Female
## 65     Nege      9A          Milk_glands          3 Negevirus  0.37158777
Female
## 67     Nege      1A      Salivary_gland          1 Negevirus  0.35831606
Female
## 71     Nege      6A                Ovary          2 Negevirus  0.35264465
Female
## 72     Nege      4A              Mid_gut          2 Negevirus  0.32100582
Female
## 79     Nege      3A Malpighian_tubules          2 Negevirus  0.15992055
Female
## 86     Nege      5A            Front_gut          2 Negevirus  0.10073370
Female
## 88     Nege      4A              Mid_gut          3 Negevirus  0.09409246
Female
## 89     Nege      2A             Hind_gut          2 Negevirus  0.08957091
Female
## 91     Nege      8A                Brain          3 Negevirus  0.05793222
Female
## 92     Nege      3A Malpighian_tubules          3 Negevirus  0.04210397
Female
## 93     Nege      5A            Front_gut          3 Negevirus  0.03995255
Female
## 94     Nege      6A                Ovary          3 Negevirus  0.03229790
Female
## 100    Nege      2A             Hind_gut          3 Negevirus  0.01216728
Female

fig1negef$Tissue <- as.factor(fig1negef$Tissue)
fig1negef$Tissue <- relevel(fig1negef$Tissue, ref= "Brain")
model1<-glm(Normalized ~ Tissue, data = fig1negef)
summary(model1)

##
## Call:
## glm(formula = Normalized ~ Tissue, data = fig1negef)
##
## Deviance Residuals:
##      Min        1Q    Median        3Q       Max
## -35.252    -0.784    -0.208     0.450    41.793
##
## Coefficients:
```

```
##                           Estimate Std. Error t value Pr(>|t|)
## (Intercept)                 1.4990     8.3066   0.180  0.85881
## TissueFat_bodies           19.2718    11.7473   1.641  0.11825
## TissueFront_gut            -1.0717    11.7473  -0.091  0.92832
## TissueHind_gut             -1.2663    11.7473  -0.108  0.91535
## TissueMalpighian_tubules    1.1008    11.7473   0.094  0.92638
## TissueMid_gut              -1.1103    11.7473  -0.095  0.92575
## TissueMilk_glands          -0.7109    11.7473  -0.061  0.95241
## TissueOvary                -1.0384    11.7473  -0.088  0.93054
## TissueSalivary_gland       34.1111    11.7473   2.904  0.00947 **
## ---
## Signif. codes:  0 '***' 0.001 '**' 0.01 '*' 0.05 '.' 0.1 ' ' 1
##
## (Dispersion parameter for gaussian family taken to be 206.997)
##
##     Null deviance: 7541.5  on 26  degrees of freedom
## Residual deviance: 3725.9  on 18  degrees of freedom
## AIC: 229.66
##
## Number of Fisher Scoring iterations: 2
```

```
Anova(model1)
```

```
## Analysis of Deviance Table (Type II tests)
##
## Response: Normalized
##        LR Chisq Df Pr(>Chisq)
## Tissue   18.433  8    0.01821 *
## ---
## Signif. codes:  0 '***' 0.001 '**' 0.01 '*' 0.05 '.' 0.1 ' ' 1
```

```
fig1negef$Tissue <- relevel(fig1negef$Tissue, ref= "Mid_gut")
model1<-glm(Normalized ~ Tissue, data = fig1negef)
summary(model1)
```

```
##
## Call:
## glm(formula = Normalized ~ Tissue, data = fig1negef)
##
## Deviance Residuals:
##     Min       1Q   Median       3Q      Max
## -35.252   -0.784   -0.208    0.450   41.793
##
## Coefficients:
##                           Estimate Std. Error t value Pr(>|t|)
## (Intercept)                0.38877    8.30656   0.047  0.96319
## TissueBrain                1.11026   11.74726   0.095  0.92575
## TissueFat_bodies          20.38208   11.74726   1.735  0.09982 .
## TissueFront_gut            0.03852   11.74726   0.003  0.99742
## TissueHind_gut            -0.15603   11.74726  -0.013  0.98955
## TissueMalpighian_tubules   2.21101   11.74726   0.188  0.85281
```

```
## TissueMilk_glands         0.39939   11.74726   0.034  0.97325
## TissueOvary               0.07186   11.74726   0.006  0.99519
## TissueSalivary_gland     35.22135   11.74726   2.998  0.00771 **
## ---
## Signif. codes:  0 '***' 0.001 '**' 0.01 '*' 0.05 '.' 0.1 ' ' 1
##
## (Dispersion parameter for gaussian family taken to be 206.997)
##
##     Null deviance: 7541.5  on 26  degrees of freedom
## Residual deviance: 3725.9  on 18  degrees of freedom
## AIC: 229.66
##
## Number of Fisher Scoring iterations: 2
```

```
Anova(model1)
```

```
## Analysis of Deviance Table (Type II tests)
##
## Response: Normalized
##        LR Chisq Df Pr(>Chisq)
## Tissue   18.433  8    0.01821 *
## ---
## Signif. codes:  0 '***' 0.001 '**' 0.01 '*' 0.05 '.' 0.1 ' ' 1
```

```
fig1negef$Tissue <- relevel(fig1negef$Tissue, ref= "Ovary")
model1<-glm(Normalized ~ Tissue, data = fig1negef)
summary(model1)
```

```
##
## Call:
## glm(formula = Normalized ~ Tissue, data = fig1negef)
##
## Deviance Residuals:
##     Min       1Q   Median       3Q      Max
## -35.252   -0.784   -0.208    0.450   41.793
##
## Coefficients:
##                             Estimate Std. Error t value Pr(>|t|)
## (Intercept)                  0.46063    8.30656   0.055  0.95639
## TissueMid_gut               -0.07186   11.74726  -0.006  0.99519
## TissueBrain                  1.03840   11.74726   0.088  0.93054
## TissueFat_bodies            20.31022   11.74726   1.729  0.10093
## TissueFront_gut             -0.03334   11.74726  -0.003  0.99777
## TissueHind_gut              -0.22790   11.74726  -0.019  0.98474
## TissueMalpighian_tubules     2.13915   11.74726   0.182  0.85754
## TissueMilk_glands            0.32753   11.74726   0.028  0.97806
## TissueSalivary_gland        35.14949   11.74726   2.992  0.00782 **
## ---
## Signif. codes:  0 '***' 0.001 '**' 0.01 '*' 0.05 '.' 0.1 ' ' 1
##
## (Dispersion parameter for gaussian family taken to be 206.997)
```

```
##
##     Null deviance: 7541.5  on 26  degrees of freedom
## Residual deviance: 3725.9  on 18  degrees of freedom
## AIC: 229.66
##
## Number of Fisher Scoring iterations: 2
```

```
Anova(model1)
```

```
## Analysis of Deviance Table (Type II tests)
##
## Response: Normalized
##        LR Chisq Df Pr(>Chisq)
## Tissue   18.433  8    0.01821 *
## ---
## Signif. codes:  0 '***' 0.001 '**' 0.01 '*' 0.05 '.' 0.1 ' ' 1
```

```
fig1negef$Tissue <- relevel(fig1negef$Tissue, ref= "Fat_bodies")
model1<-glm(Normalized ~ Tissue, data = fig1negef)
summary(model1)
```

```
##
## Call:
## glm(formula = Normalized ~ Tissue, data = fig1negef)
##
## Deviance Residuals:
##     Min       1Q   Median       3Q      Max
## -35.252   -0.784   -0.208    0.450   41.793
##
## Coefficients:
##                            Estimate Std. Error t value Pr(>|t|)
## (Intercept)                  20.771      8.307   2.501   0.0223 *
## TissueOvary                 -20.310     11.747  -1.729   0.1009
## TissueMid_gut               -20.382     11.747  -1.735   0.0998 .
## TissueBrain                 -19.272     11.747  -1.641   0.1183
## TissueFront_gut             -20.344     11.747  -1.732   0.1004
## TissueHind_gut              -20.538     11.747  -1.748   0.0974 .
## TissueMalpighian_tubules    -18.171     11.747  -1.547   0.1393
## TissueMilk_glands           -19.983     11.747  -1.701   0.1061
## TissueSalivary_gland         14.839     11.747   1.263   0.2226
## ---
## Signif. codes:  0 '***' 0.001 '**' 0.01 '*' 0.05 '.' 0.1 ' ' 1
##
## (Dispersion parameter for gaussian family taken to be 206.997)
##
##     Null deviance: 7541.5  on 26  degrees of freedom
## Residual deviance: 3725.9  on 18  degrees of freedom
## AIC: 229.66
##
## Number of Fisher Scoring iterations: 2
```

```
Anova(model1)

## Analysis of Deviance Table (Type II tests)
##
## Response: Normalized
##        LR Chisq Df Pr(>Chisq)
## Tissue   18.433  8    0.01821 *
## ---
## Signif. codes:  0 '***' 0.001 '**' 0.01 '*' 0.05 '.' 0.1 ' ' 1

fig1negef$Tissue <- relevel(fig1negef$Tissue, ref= "Salivary_gland")
model1<-glm(Normalized ~ Tissue, data = fig1negef)
summary(model1)

##
## Call:
## glm(formula = Normalized ~ Tissue, data = fig1negef)
##
## Deviance Residuals:
##     Min       1Q   Median       3Q      Max
## -35.252   -0.784   -0.208    0.450   41.793
##
## Coefficients:
##                            Estimate Std. Error t value Pr(>|t|)
## (Intercept)                  35.610      8.307   4.287 0.000444 ***
## TissueFat_bodies            -14.839     11.747  -1.263 0.222631
## TissueOvary                 -35.149     11.747  -2.992 0.007818 **
## TissueMid_gut               -35.221     11.747  -2.998 0.007715 **
## TissueBrain                 -34.111     11.747  -2.904 0.009468 **
## TissueFront_gut             -35.183     11.747  -2.995 0.007770 **
## TissueHind_gut              -35.377     11.747  -3.012 0.007495 **
## TissueMalpighian_tubules    -33.010     11.747  -2.810 0.011585 *
## TissueMilk_glands           -34.822     11.747  -2.964 0.008306 **
## ---
## Signif. codes:  0 '***' 0.001 '**' 0.01 '*' 0.05 '.' 0.1 ' ' 1
##
## (Dispersion parameter for gaussian family taken to be 206.997)
##
##     Null deviance: 7541.5  on 26  degrees of freedom
## Residual deviance: 3725.9  on 18  degrees of freedom
## AIC: 229.66
##
## Number of Fisher Scoring iterations: 2

Anova(model1)

## Analysis of Deviance Table (Type II tests)
##
## Response: Normalized
##        LR Chisq Df Pr(>Chisq)
## Tissue   18.433  8    0.01821 *
```

```
## ---
## Signif. codes:  0 '***' 0.001 '**' 0.01 '*' 0.05 '.' 0.1 ' ' 1

fig1negef$Tissue <- relevel(fig1negef$Tissue, ref= "Front_gut")
model1<-glm(Normalized ~ Tissue, data = fig1negef)
summary(model1)

##
## Call:
## glm(formula = Normalized ~ Tissue, data = fig1negef)
##
## Deviance Residuals:
##     Min       1Q   Median       3Q      Max
## -35.252   -0.784   -0.208    0.450   41.793
##
## Coefficients:
##                            Estimate Std. Error t value Pr(>|t|)
## (Intercept)                 0.42729    8.30656   0.051  0.95954
## TissueSalivary_gland       35.18283   11.74726   2.995  0.00777 **
## TissueFat_bodies           20.34356   11.74726   1.732  0.10042
## TissueOvary                 0.03334   11.74726   0.003  0.99777
## TissueMid_gut              -0.03852   11.74726  -0.003  0.99742
## TissueBrain                 1.07173   11.74726   0.091  0.92832
## TissueHind_gut             -0.19456   11.74726  -0.017  0.98697
## TissueMalpighian_tubules  2.17249   11.74726   0.185  0.85535
## TissueMilk_glands           0.36086   11.74726   0.031  0.97583
## ---
## Signif. codes:  0 '***' 0.001 '**' 0.01 '*' 0.05 '.' 0.1 ' ' 1
##
## (Dispersion parameter for gaussian family taken to be 206.997)
##
##     Null deviance: 7541.5  on 26  degrees of freedom
## Residual deviance: 3725.9  on 18  degrees of freedom
## AIC: 229.66
##
## Number of Fisher Scoring iterations: 2

Anova(model1)

## Analysis of Deviance Table (Type II tests)
##
## Response: Normalized
##        LR Chisq Df Pr(>Chisq)
## Tissue   18.433  8    0.01821 *
## ---
## Signif. codes:  0 '***' 0.001 '**' 0.01 '*' 0.05 '.' 0.1 ' ' 1

fig1negef$Tissue <- relevel(fig1negef$Tissue, ref= "Hind_gut")
model1<-glm(Normalized ~ Tissue, data = fig1negef)
summary(model1)
```

```
##
## Call:
## glm(formula = Normalized ~ Tissue, data = fig1negef)
##
## Deviance Residuals:
##     Min       1Q   Median       3Q      Max
## -35.252   -0.784   -0.208    0.450   41.793
##
## Coefficients:
##                           Estimate Std. Error t value Pr(>|t|)
## (Intercept)                 0.2327     8.3066   0.028  0.97796
## TissueFront_gut             0.1946    11.7473   0.017  0.98697
## TissueSalivary_gland       35.3774    11.7473   3.012  0.00749 **
## TissueFat_bodies           20.5381    11.7473   1.748  0.09744 .
## TissueOvary                 0.2279    11.7473   0.019  0.98474
## TissueMid_gut               0.1560    11.7473   0.013  0.98955
## TissueBrain                 1.2663    11.7473   0.108  0.91535
## TissueMalpighian_tubules    2.3670    11.7473   0.201  0.84257
## TissueMilk_glands           0.5554    11.7473   0.047  0.96281
## ---
## Signif. codes:  0 '***' 0.001 '**' 0.01 '*' 0.05 '.' 0.1 ' ' 1
##
## (Dispersion parameter for gaussian family taken to be 206.997)
##
##     Null deviance: 7541.5  on 26  degrees of freedom
## Residual deviance: 3725.9  on 18  degrees of freedom
## AIC: 229.66
##
## Number of Fisher Scoring iterations: 2
```

```
Anova(model1)
```

```
## Analysis of Deviance Table (Type II tests)
##
## Response: Normalized
##        LR Chisq Df Pr(>Chisq)
## Tissue   18.433  8    0.01821 *
## ---
## Signif. codes:  0 '***' 0.001 '**' 0.01 '*' 0.05 '.' 0.1 ' ' 1
```

```
fig1negef$Tissue <- relevel(fig1negef$Tissue, ref= "Milk_glands")
model1<-glm(Normalized ~ Tissue, data = fig1negef)
summary(model1)
```

```
##
## Call:
## glm(formula = Normalized ~ Tissue, data = fig1negef)
##
## Deviance Residuals:
##     Min       1Q   Median       3Q      Max
## -35.252   -0.784   -0.208    0.450   41.793
```

```
##
## Coefficients:
##                            Estimate Std. Error t value Pr(>|t|)
## (Intercept)                  0.7882     8.3066   0.095  0.92546
## TissueHind_gut              -0.5554    11.7473  -0.047  0.96281
## TissueFront_gut             -0.3609    11.7473  -0.031  0.97583
## TissueSalivary_gland        34.8220    11.7473   2.964  0.00831 **
## TissueFat_bodies            19.9827    11.7473   1.701  0.10614
## TissueOvary                 -0.3275    11.7473  -0.028  0.97806
## TissueMid_gut               -0.3994    11.7473  -0.034  0.97325
## TissueBrain                  0.7109    11.7473   0.061  0.95241
## TissueMalpighian_tubules     1.8116    11.7473   0.154  0.87915
## ---
## Signif. codes:  0 '***' 0.001 '**' 0.01 '*' 0.05 '.' 0.1 ' ' 1
##
## (Dispersion parameter for gaussian family taken to be 206.997)
##
##     Null deviance: 7541.5  on 26  degrees of freedom
## Residual deviance: 3725.9  on 18  degrees of freedom
## AIC: 229.66
##
## Number of Fisher Scoring iterations: 2
```

```
Anova(model1)
```

```
## Analysis of Deviance Table (Type II tests)
##
## Response: Normalized
##        LR Chisq Df Pr(>Chisq)
## Tissue   18.433  8    0.01821 *
## ---
## Signif. codes:  0 '***' 0.001 '**' 0.01 '*' 0.05 '.' 0.1 ' ' 1
```

```
fig1negef$Tissue <- relevel(fig1negef$Tissue, ref=
"Malpighian_tubules")
model1<-glm(Normalized ~ Tissue, data = fig1negef)
summary(model1)
```

```
##
## Call:
## glm(formula = Normalized ~ Tissue, data = fig1negef)
##
## Deviance Residuals:
##     Min       1Q   Median       3Q      Max
## -35.252   -0.784   -0.208    0.450   41.793
##
## Coefficients:
##                        Estimate Std. Error t value Pr(>|t|)
## (Intercept)               2.600      8.307   0.313   0.7579
## TissueMilk_glands        -1.812     11.747  -0.154   0.8792
## TissueHind_gut           -2.367     11.747  -0.201   0.8426
```

```
## TissueFront_gut        -2.172     11.747  -0.185   0.8553
## TissueSalivary_gland   33.010     11.747   2.810   0.0116 *
## TissueFat_bodies       18.171     11.747   1.547   0.1393
## TissueOvary            -2.139     11.747  -0.182   0.8575
## TissueMid_gut          -2.211     11.747  -0.188   0.8528
## TissueBrain            -1.101     11.747  -0.094   0.9264
## ---
## Signif. codes:  0 '***' 0.001 '**' 0.01 '*' 0.05 '.' 0.1 ' ' 1
##
## (Dispersion parameter for gaussian family taken to be 206.997)
##
##     Null deviance: 7541.5  on 26  degrees of freedom
## Residual deviance: 3725.9  on 18  degrees of freedom
## AIC: 229.66
##
## Number of Fisher Scoring iterations: 2
```

```
Anova(model1)
```

```
## Analysis of Deviance Table (Type II tests)
##
## Response: Normalized
##        LR Chisq Df Pr(>Chisq)
## Tissue   18.433  8    0.01821 *
## ---
## Signif. codes:  0 '***' 0.001 '**' 0.01 '*' 0.05 '.' 0.1 ' ' 1
```
